# ﻿A review of cave spiders (Arachnida, Araneae) of the Crimean Mountains, with descriptions of two new species

**DOI:** 10.3897/zookeys.1230.137029

**Published:** 2025-03-05

**Authors:** Anton A. Nadolny, Ilya S. Turbanov

**Affiliations:** 1 A.O. Kovalevsky Institute of Biology of the Southern Seas, Nakhimov Ave. 2, Sevastopol 299011 A.O. Kovalevsky Institute of Biology of the Southern Seas Sevastopol Ukraine; 2 Papanin Institute for Biology of Inland Waters Russian Academy of Sciences, Borok 152742, Yaroslavl Region, Russia Papanin Institute for Biology of Inland Waters Russian Academy of Sciences Borok Russia; 3 Cherepovets State University, Lunacharskogo Ave. 5, Cherepovets 162600, Vologda Region, Russia Cherepovets State University Cherepovets Russia

**Keywords:** Aranei, faunistics, subterranean biology, taxonomy, troglobiont

## Abstract

Based on a critical review of the literature and study of the authors’ own collections, a survey of cave spiders of the Crimean Mountains has been conducted, resulting in 20 reliable species records in eight families. Nine species have been discovered in the Crimean caves for the first time, of which two are described as new to science. A classification of spiders by ecological groups depending on their cave lifestyle is provided; a troglomorphic spider is found and described from Crimea for the first time. The most likely scenarios of spider colonization into underground habitats of Crimea are discussed. Most species arrived during multiple Pleistocene-Holocene regressions of the Black Sea basin, when zoogeographic corridors on the exposed shelf connected Crimea with the Caucasus and the Balkans. However, four synanthropic species entered Crimean caves in historical times. High relative humidity and temperature are considered key factors that enable caves to serve as refugia for Pleistocene araneofauna. A zoogeographical analysis of cave spiders is carried out. The majority of the spider species considered, totalling 16 species, are widespread, with ranges including cosmopolitan, Holarctic, trans-Palaearctic, West and Central Palaearctic, East European, and East Mediterranean. Three species are endemic to Crimea: *Tegenariataurica*, *Bisetifertactus***sp. nov.**, and *Troglohyphantesexspectatus***sp. nov.***Bisetifergruzin* is a Crimean-Caucasian subendemic species.

## ﻿Introduction

The study of the subterranean fauna of the Crimean Peninsula has more than a centennial history (Turbanov et al. 2016a, b, c). The first data on cave spiders in the region in question appeared in the papers by Lebedinsky (1904, 1914), Novikov (1912) and Spassky (1927, 1936). Later, Charitonov (1947a) re-examined the material of Lebedinsky (1904, 1914) and revealed that many of his records were based on misidentifications; he also described two new species: *Tegenariataurica* Charitonov, 1947 (Agelenidae) and *Palliduphanteskhobarum* (Charitonov, 1947) (Linyphiidae). All subsequent reports on the CrImean cave spiders have been based on occasional collections (Evtushenko 2004; Zagorodniuk and Vargovitsh 2004; Kovblyuk 2004b, 2007; Nadolny and Turbanov 2014; Turbanov et al. 2019a, b, 2021). In total, there are 14 publications that have provided 11 spider species for the Crimean caves. Compared to other karst regions of the Alpine-Mediterranean foldbelt, the Crimean caves are characterised by lower spider diversity (Turbanov et al. 2016b; Mammola et al. 2018). Yet, the overall spider diversity in Crimea is equal/comparable to that of neighbouring regions of similar size (Kovblyuk and Kastrygina 2015; Nentwig et al. 2024). The level of regional species endemism remains quite low, with only 11 spider species that are confined to Crimea, including one cave species, *T.taurica*. At the same time, two monotypic endemic genera, *Deliriosa* Kovblyuk, 2009 (Lycosidae) and *Spinestis* Saaristo & Marusik, 2009 (Oonopidae), are known from the mountainous part of the peninsula (Kovblyuk 2014; see WSC 2024 for additional records). The endemism at the generic level indicates that the age of the araneofauna in the Crimean Mountains could be comparable to those of the Balkans (14 endemic genera, including cave genera) and the Caucasus (one endemic genus from an endemic subfamily; see Kovblyuk 2014).

Thus, compared to the well-studied epigean araneofauna of the Crimean Peninsula, subterranean spiders are still poorly studied. For this reason, the first thorough survey of the Crimean cave spiders is the main objective of the present paper, aiming at (1) a critical analysis of literature-derived data; (2) providing new faunistic and taxonomic data; (3) presenting a possible ecological classification of the Crimean cave species depending on their association with caves; (4) discussing the possible scenarios of spider penetration into the Crimean underground habitats; and (5) undertaking a zoogeographic analysis of the Crimean spider fauna.

## ﻿Materials and methods

The material for the present study has been hand-collected from 31 the Crimean caves over the decade 2010–2021. A total of 243 spider specimens (216 adults, 5 subadults and 22 juveniles) belonging to 20 species have been collected and identified; two additional species (two specimens) were studied as comparative material. All the material was preserved in 96% alcohol directly in the caves.

For the study, the copulatory organs were dissected, boiled in a 10% aqueous solution of potassium hydroxide (KOH), and placed in glycerine on a slide with a cavity. The photos of copulatory organs and general appearances were taken using Canon EOS 550D camera mounted on MBS-1 and Olympus CX41 microscopes and a Hitachi SU3500 scanning electron microscope at the A.O. Kovalevsky Institute of Biology of the Southern Seas (Sevastopol). Resulting images were processed in the Helicon Focus 7.0.2., Adobe Photoshop CS6 and CorelDRAW 11 programs. Some maps were created using Google Earth Pro version 7.3.0.3832 and Adobe Photoshop CS6.

Types and voucher specimens have been shared between the Zoological Museum of the Moscow State University, Russia (**ZMMU**); the National Arachnological Collection, the V.I. Vernadsky Tavrida National University (“Crimean Federal University”), Simferopol, Crimea (**TNU**); and the private collection of the second author (**IT**).

When discussing ecological groups of the Crimean cave spiders, the classification by Sket (2008) has been adopted: (1) troglobiont, i.e., a species/population strictly that is bound to a hypogean habitats; (2) eutroglophile, i.e., an essentially epigeic species that is capable of maintaining a permanent subterranean population; (3) subtroglophile, i.e. inclined perpetually/temporarily to inhabiting in subterranean habitats, but requires the surface for some biological functions (e.g., feeding); (4) trogloxene, i.e., a species that only sporadically (accidentally) becomes subterranean.

## ﻿Results

**Class Arachnida Lamarck, 1801**

**Order Araneae Clerck, 1757**

### ﻿Family Agelenidae C.L. Koch, 1837

#### ﻿Genus *Tegenaria* Latreille, 1804

##### ﻿ Tegenaria lapicidinarum

Taxon classificationAnimaliaAraneaeAgelenidae

Spassky, 1934

16C19A9B-A961-5477-A6E3-687D9C8F01F2

Fig. 1A

Tegenaria lapicidinarum Spassky, 1934: Evtushenko 2004: 66–68; Zagorodniuk and Vargovitsh 2004: 207; Kovblyuk 2014: 44; Turbanov et al. 2016b: 1283.

###### Material examined.

• 1 ♀ (TNU 10189), Crimea, Simferopol Distr., central part of Tshatyr-Dagh Yaila, nr Vyalovsky Forest, Alushtinskaya Cave, 11.II.2015, I.S. Turbanov leg. • 3 ♀♀ (TNU 10187), Crimea, Belogorsk Distr., northeastern part of Karabi Yaila, Karani-Koba Cave, 29.I.2014, I.S. Turbanov leg.

###### Distribution.

East European nemoral: Ukraine and the south part of European Russia. The Crimea represents the southernmost limit of the species range (Kovblyuk 2004b; Kovblyuk and Kastrygina 2015; Nentwig et al. 2024).

###### Records from the Crimean caves.

Map (Fig. 17A – purple circle). Unnamed cave near the city of Bakhchisarai, Alushtinskaya Сave in Tshatyr-Dagh Yaila, and Karani-Koba Cave in Karabi Yaila (Evtushenko 2004; present data).

###### Ecology.

A troglophile (Evtushenko 2004; Zagorodniuk and Vargovitsh 2004). In Crimea, *T.lapicidinarum* is a common species, occurring in all landscape zones from the seashore to yaila (Kovblyuk 2004b). In addition to the Crimean subterranean biotopes, this species has also been recorded from catacombs of Odessa, Ukraine (Deli et al. 2017). This species is common in terrestrial habitats, but is rarely found in caves, and so is here classified as a subtroglophile.

##### ﻿ Tegenaria parietina

Taxon classificationAnimaliaAraneaeAgelenidae

(Fourcroy, 1785)

1D688E5E-5AFB-5F0F-A153-03577D4070B1

###### Material examined.

• 1 ♀ (TNU 10190/1), Crimea, nr Sevastopol, Khomutovaya Gorge, nr Maksimovа Datsha, abandoned aqueduct carved into an unnamed cave-spring, 23.V.2015, A.A. Nadolny leg.

###### Distribution.

Cosmopolite (Kovblyuk and Kastrygina 2015; Nentwig et al. 2024).

###### Records from the Crimean caves.

Map (Fig. 17A – blue circle). Abandoned aqueduct carved into an unnamed cave-spring nr Maksimova Datsha, Sevastopol (present data).

###### Ecology.

A troglophile and synanthropic species (Mammola et al. 2018; Nentwig et al. 2024). In Crimea, *T.parietina* inhabits mountainous and foothill areas (Kovblyuk and Kastrygina 2015), and has not been previously recorded from the Crimean caves. However, during our surveys of subterranean the Crimean biotopes, we have once found this species in the abandoned aqueduct of Maksimova Datsha – the site of intensive agricultural and other economic activities in the second half of the 19^th^ and early 20^th^ centuries (Chikin 2005). For this reason, we believe that *T.parietina* is not a permanent member of the Crimean cave fauna. This species is likely to be a facultative synanthrope that can inhabit underground biotopes as a subtroglophile.

##### ﻿ Tegenaria taurica

Taxon classificationAnimaliaAraneaeAgelenidae

Charitonov, 1947

D814A716-6592-5C97-B8D1-9E1C031F3FFD

Figs 1B , 3 , 4 , 5

Tegenaria taurica Charitonov, 1947: Charitonov 1947a: 44–49, 51, 54, figs 4, 5; Charitonov 1947b: 1; Birstein 1963: 128; Tyshchenko 1971: 23, 156, 161, 163; Mikhailov 1997: 145; Mikhailov 1998: 22; Esyunin and Farzalieva 2001: 261–263, figs 1–5; Kovblyuk 2002: 105; Amelichev et al. 2004: 136, 140; Evtushenko 2004: 66, 68; Kovblyuk 2004a: 214; Kovblyuk 2004b: 43, 45, 47–48, figs 2, 3(1); Kovblyuk 2004c: 254, 256; Zagorodniuk and Vargovitsh 2004: 207; Kovblyuk 2010: 224; Mikhailov 2013: 141; Bolzern et al. 2013: 776, 803, 818, 846; Kovblyuk 2014: 34, 44, 51; Kovblyuk and Kastrygina 2015: 6; Turbanov et al. 2016b: 1283; Prokopov and Turbanov 2017: 101; Mammola et al. 2018: table S1; Samokhin et al. 2019: 247.
Tegenaria domestica (Clerck, 1757): Kovblyuk 2014: 44.
Tegenaria civilis Walk. [sic!]: Lebedinsky 1904: 77.
Tegenaria derhami (Scopoli, 1763): Charitonov 1932: 21; Charitonov 1939: 197.
Tegenaria sp.: Evtushenko 2004: 67; Zagorodniuk and Vargovitsh 2004: 208; Turbanov et al. 2019a: 218.
Meta menardi (Latreille, 1804): Lebedinsky 1914: 115, 117, 121–122; Charitonov 1932: 123; Charitonov 1939: 197; Tyshchenko 1971: 190; Mikhailov 1997: 108; Kovblyuk 2004a: 245; Mikhailov 2013: 101; Kovblyuk 2014: 44; Kovblyuk and Kastrygina 2015: 56.

###### Material examined.

• 2 ♂♂ (TNU 10260), Crimea, Sevastopol, nr Balaklava, southern slope of Mt. Asketi, Asketi I Cave, 26.IX.2015, O.V. Kukushkin leg. • 7 ♀♀ (TNU 10259/1), Crimea, Sevastopol, nr Balaklava, Aya Cape Mt. Range, Mt. Kala-Fatlar, Izumrudnaya Cave, 20.III.2016, O.V. Kukushkin leg. • 1 ♀ (TNU 10180/1), Crimea, Sevastopol, nr Balaklava, Aya Cape Mt. Range, Mt. Kala-Fatlar, Gekkonovaya Cave, 09.X.2016, A.A. Nadolny leg. • 1 ♀ (TNU 10287/1), Crimea, Sevastopol, Tshernaya River canyon, Tshernoretshenskaya Cave, 3.VI.2021, I.S. Turbanov, A.A. Nadolny leg. • 1 ♀ (IT), Crimea, Sevastopol, northeastern slope of Baidarskаya Yaila, Baidarskaya Valley, nr Kizilovoye Vil., Mamut-Tshokrak Cave, 26.VII.2010, I.S. Turbanov leg. • 1 ♀ (TNU 10257/3), 1 ♀ (IT), Crimea, Sevastopol, northwestern slope of Ai-Petri Yaila, Baidarskaya Valley, nr Rodnikovskoye Vil., entrance to Skelskaya Cave, 29.IX.2020, A.A. Nadolny leg. • 2 ♀♀ (TNU 10197), Crimea, Sevastopol, NW slope of Ai-Petri Yaila, nr Karadagh Forest, Rodnikovskaya Cave, 4.II.2014, I.S. Turbanov leg. • 3 ♀♀ (TNU 10196), Crimea, Sevastopol, northwestern slope of Ai-Petri Yaila, nr Karadagh Forest, Koryta (= Kuznetsova) Cave, 8.III.2014, I.S. Turbanov leg.• 1 ♀ (IT), Crimea, Sevastopol, southwestern part of Ai-Petri Yaila, Mortsheka Mt., Druzhba Cave, 3.X.2020, I.S. Turbanov leg. • 1 ♀ (TNU 10261/1), Crimea, Bakhchisarai Distr., northern part of Ai-Petri Yaila, Mt. Ayu-Teshik, Ayu-Teshik Cave, 8.V.2015, I.S. Turbanov leg. • 1 ♀ (TNU 10227), Crimea, Bakhchisarai Distr., nr Stshastlivoe Vil., northwestern slope of Yalta Yaila, Khaplu-Kaya Mt., Kaply-Kayanskaya (Khaplu-Khoba) Cave, 29.VI.2017, I.S. Turbanov leg. • 1 ♀ (TNU 10195/1), Crimea, Simferopol Distr., northern part of Tshatyr-Dagh Yaila, Binbash-Koba Cave, 12.II.2015, I.S. Turbanov leg. • 1 ♀ (IT), Crimea, Simferopol Distr., nr Perevalnoye Vil., western slope of Dolgorukovskaya Yaila, Kizil-Koba (= Krasnaya) Cave, 5.XI.2014, I.S. Turbanov leg.

**Figure 1.**
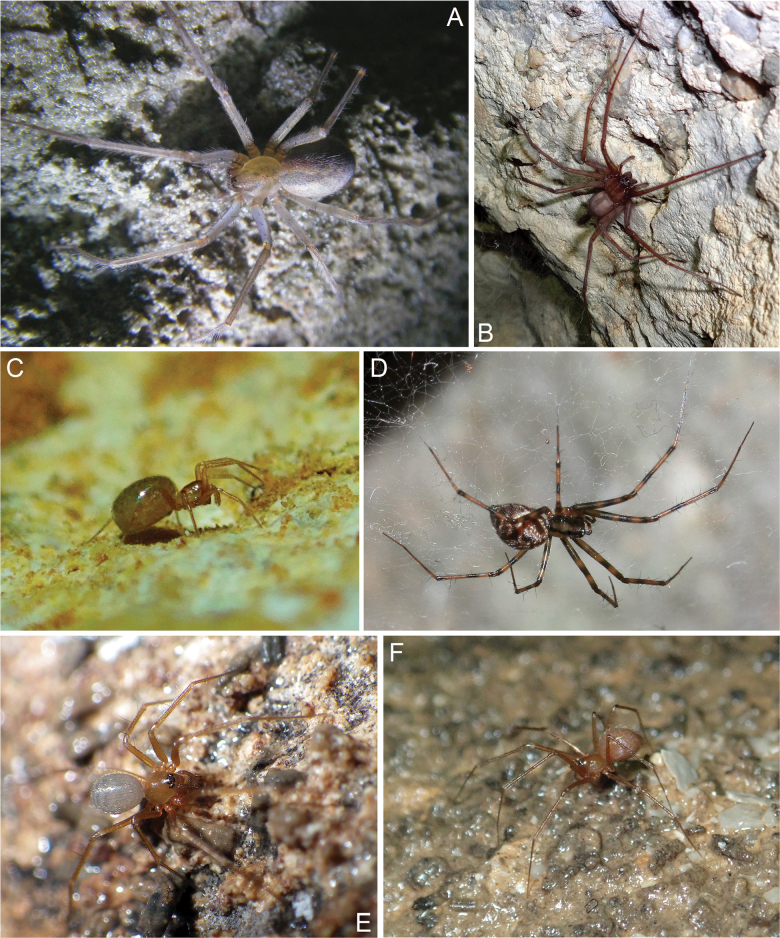
Spiders in situ in caves of Crimea: **A***Tegenarialapicidinarum*, ♀ from Karani-Koba Cave **B***Tegenariataurica*, ♀ from Gekkonovaya Cave **C***Caviphantesdobrogicus*, ♀ from Tavrida Cave **D***Megalepthyphantespseudocollinus*, ♀ from Skelskaya Cave **E***Palliduphanteskhobarum*, ♀ from Skelskaya Cave **F***Troglohyphantesexspectatus* sp. nov., ♀ from Druzhba Cave. Photographs by IST (**A, C, F**); AAN (**B, D**); G.A. Prokopov (**E**).

**Figure 2.**
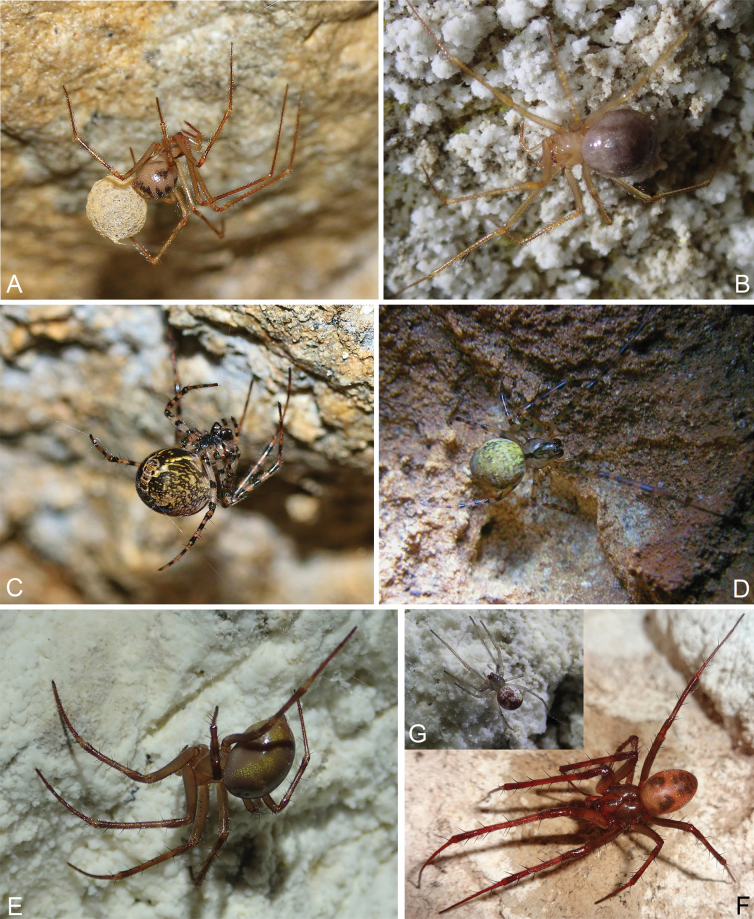
Spiders in situ in caves of Crimea: **A***Aituariapontica*, ♀ from abandoned aqueduct carved into an unnamed cave-spring in area of the Maksimova Datsha in the nr Sevastopol **B***Aituariaborutzkyi*, ♀ from Mangupskaya I Cave **C, D***Metellinamerianae*, ♀♀ from abandoned aqueduct carved into an unnamed cave-spring in area of the Maksimova Datsha in the nr Sevastopol (**С**) and Mamut-Tshokrak Cave (**D**); **E**, **F**, **G***Metabourneti*, ♀ (**E**) and juv. (**G**) from from Mangupskaya I Cave, ♂ (**F**) from Gnomov Cave. Photographs by G.A. Prokopov (**A, C**); IST (**B, D, E, G**); AAN (**F**).

###### Distribution.

Endemic of the Crimean Mountains (Kovblyuk and Kastrygina 2015). However, there is a dubious record from Georgia (Mkheidze 1997), which has never been confirmed by any collected material (Kovblyuk and Kastrygina 2015).

###### Records from the Crimean caves.

Map (Fig. 17A – orange circle). *Tegenariataurica* is known from caves in the western and central parts of the Crimean Mountains: small unnamed cave (= ?Malaya Cave) in Nizhnie Limeny (now Goluboi Zaliv, region of Yalta; the locality for male syntype *T.taurica* – sensu Charitonov 1947a), Asketi I, Izumrudnaya, Gekkonovaya, Tshernoretshenskaya, Mamut-Tshokrak, Skelskaya, Rodnikovskaya, Koryta, Druzhba, Ayu-Teshik (= Ayutishik-Koba; the locality for female syntype *T.taurica* – sensu Charitonov 1947a), Daniltsha-Koba, Kaply-Kayanskaya, Ayu-Koba, Binbash-Koba, Kizil-Koba, and grotto in Massandra (the type locality as that of the lectotype – sensu Esyunin and Farzalieva 2001), grotto on Mt. Yuznaya Demerdzhi (Lebedinsky 1904, 1914; Charitonov 1947a; Esyunin and Farzalieva 2001; Kovblyuk 2004b; Samokhin et al. 2019; Turbanov et al. 2019a; present data).

###### Ecology.

A troglophile (Mammola et al. 2018). There is a single record of *T.taurica* from an anthropogenic biotope (Kovblyuk 2004b), which in fact refers to *T.parietina* (1 ♂ (TNU 1630/1), Yalta, indoors, 17.X.2001 – examined), the remaining findings have been from caves (present data). Thus, this species is here referred to as eutroglophile.

###### Remarks.

According to Charitonov (1947a, b), who described *T.taurica* on the basis of the collection of spiders reported earlier by Lebedinsky (1904, 1914), the earlier records of *T.civilis*, *T.derhami* and partly of *Metamenardi* from the Crimean caves (Lebedinsky 1904, 1914; Charitonov 1932, 1939) should in fact be assigned to *T.taurica*. Yet, a number of researchers, although with doubt, have continued to erroneously report on *M.menardi* for the Crimean caves (Tyshchenko 1971; Mikhailov 1997; Kovblyuk 2004a, 2014; Mikhailov 2013; Kovblyuk and Kastrygina 2015). The report on *T.domestica* in the Crimean caves is erroneous (Kovblyuk 2014), as it was based on the record of *T.civilis* by Lebedinsky (1904), and actually belongs to *T.taurica* (M.M. Kovblyuk, pers. comm.). The records of *Tegenaria* sp. from Mamut-Tshokrak Cave (Turbanov et al. 2019a) and Kizil-Koba Cave (Evtushenko 2004) should also be assigned to *T.taurica*, which has been confirmed by the present study (see Material examined).

*Tegenariataurica* was redescribed by Esyunin and Farzalieva (2001), who also designated the lectotype based on the material from the Massandra grotto nr Yalta. Among the paralectotypes there is the specimen labelled as follows: “1 slide preparation of palp (PSU), Crimea, Nizhnie Limeny, Malaya Caves, 08–22.IX.1916, leg. L.A. Lants”. The male used for the description of *T.taurica* was collected from the same cave (see Charitonov 1947a: 47, 51).

The mention of *T.taurica* for Tuakskaya (= Ful-Koba) Cave was presumably based on the erroneous label “Ayu-Tishik-Koba. Tuvak. Meta spes? vois. de Menardi” (see Charitonov 1947a: 45) and “Ayutishik-Koba [caves], Tuvak, 1905, leg. Ya.N. Lebedinskii” (see Esyunin and Farzalieva 2001: 261), when two different caves are mistakenly indicated: viz., Ayu-Teshik (as Ayu-Tishik-Koba and Ayutishik-Koba) and Tuakskaya (as Tuvak), whereas they are situated in different parts of the Crimean Mountains. However, in the original work by Lebedinsky (1914), *T.taurica* (as *Metamenardi*) is recorded from Ayu-Teshik Cave, and *Palliduphanteskhobarum* (as *Lephthyphantes* [sic!] *monticola*) from Tuakskaya Cave. Our repeated survey in Tuakskaya Cave has confirmed that the only spider species occurring there is *P.khobarum*.

**Figure 3.**
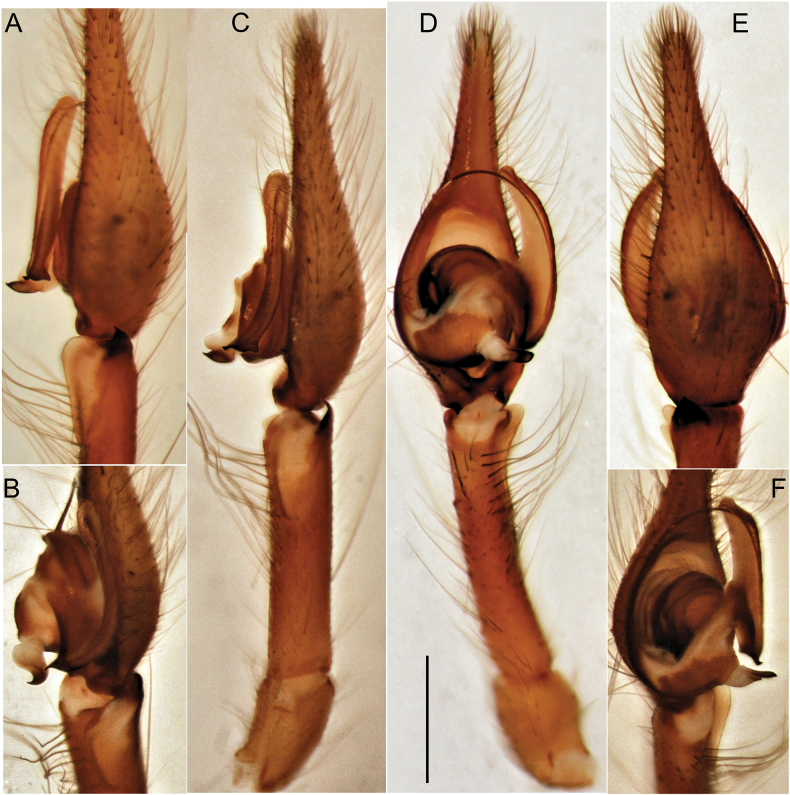
Male palp of *Tegenariataurica* from Asketi I Cave: **A** dorso-retrolateral view **B** anterio-retrolateral view **C** retrolateral view **D** ventral view **E** dorsal view **F** ventro-prolateral view. Scale bar: 1.0 mm.

**Figure 4.**
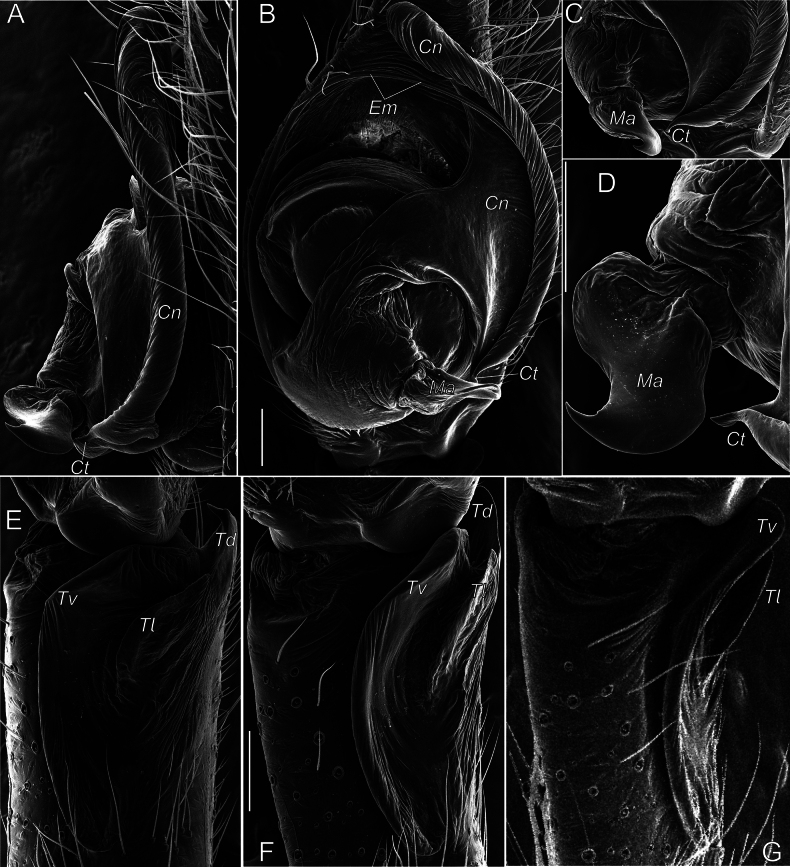
Male palp of *Tegenariataurica* from Asketi I Cave: **A, B** bulbus, retrolateral and ventral views **C, D** fragment of bulbus with median apophysis and tip of conductor, anterio-retrolateral and anterior views **E–G** tibial apophyses, retrolateral, ventro-retrolateral and ventral views. Abbreviations: *Cn* – conductor, *Ct* – tip of conductor, *Em* – embolus, *Ma* – median apophysis, *Td* – dorsal tibial apophysis, *Tl* – lateral tibial apophysis, *Tv* – ventral tibial apophysis. Scale bars: 0.2 mm.

**Figure 5.**
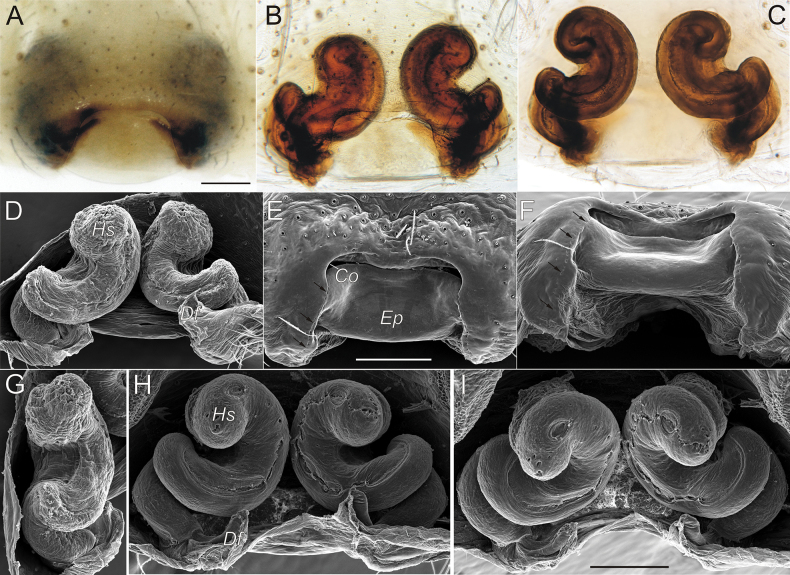
Epigynes of *Tegenariataurica* from Kuznetsova Cave: **A, B, E** ventral view **C, D, H** dorsal view **F** posterior view **G** lateral view **I** anterior view. Abbreviations: *Co* – copulatory opening, *Df* – fertilization duct, *Ep* – epigynal plate, *Hs* – head of spermatheca. Arrows indicate borders of epigynal plate. Scale bars: 0.2 mm.

We consider it appropriate to provide an illustrated description of the copulatory organs of both sexes of *T.taurica* from the Crimean caves (Figs 3–5). The cymbium and tibia+patella lengths are equal (Fig. 3С). Tibia has three apophyses (Fig. 4E–G): dorsal apophysis pointed and well sclerotised (Fig. 4A, C, E), ventral and lateral – rounded and poorly sclerotised (Fig. 4A, D, E); embolus originated at 320° position and terminating at about 210° position, makes an ellipsoid trajectory and holds it distal part in conductor (Figs 3D, F, 4B); conductor with two arms in longitudinal position; embolic and conductor tips directed posteriorly (Figs 3A–C, 4A, B); median apophysis flat with sharped tip directed ventrally (Figs 3B, 4C, D). Epigyne with trapezoid plate (Fig. 5A); lateral borders of epigynal plate poorly recognised (Fig. 5E, F); spermatheca massive, makes two curves – ventral and sagittal (Fig. 5C, G, I); head of spermatheca variable (Fig. 5D, H).

### ﻿Family Amaurobiidae Thorell, 1869

#### ﻿Genus *Amaurobius* C.L. Koch, 1837

##### ﻿ Amaurobius erberi

Taxon classificationAnimaliaAraneaeAmaurobiidae

(Keyserling, 1863)

D20742D1-A40C-54F4-ACEB-8B852903B6E7

###### Material examined.

• 1 ♂ (TNU 10237/2), Crimea, Bakhchisarai Distr., nr Khodzha-Sala Vil., steep southern slope of Baba-Dagh Plateau (= Mangup-Kale Gorodishche), Mangupskaya I (= MK-1) Cave, 3.V.2018, I.S. Turbanov leg.

###### Distribution.

West Palearctic nemoral-subtropical: from the Canaries to Azerbaijan and from Central Europe to Algeria (Kovblyuk and Kastrygina 2015; Nentwig et al. 2024).

###### Records from the Crimean caves.

Map (Fig. 17A – yellow circle). Mangupskaya I Cave on steep southern slope of Baba-Dagh Plateau (present data).

###### Ecology.

In Crimea, *Amaurobiuserberi* is widespread and found in all landscape zones (Kovblyuk and Kastrygina 2015). This species has not been previously recorded from caves and it is hardly a permanent cave dweller, so it can be classified as a trogloxene.

### ﻿Family Linyphiidae Blackwall, 1859

#### ﻿Genus *Bisetifer* Tanasevitch, 1987

##### ﻿ Bisetifer gruzin

Taxon classificationAnimaliaAraneaeLinyphiidae

Tanasevitch, Ponomarev & Chumachenko, 2015

DBA62031-8A5C-577D-A411-60A516238B14

Fig. 6C, D

Bisetifer cephalotus Tanasevitch, 1987: Kovblyuk 2007: 152; Mikhailov 2013: 45; Tanasevitch et al. 2015: 445–446.

###### Material examined.

• 1 subad. ♂, 1 ♀ (TNU 10288), Crimea, Simferopol Distr., nr Perevalnoye Vil., western slope of Dolgorukovskaya Yaila, Kizil-Koba (= Krasnaya) Cave, 18.XII.2019, I.S. Turbanov leg.

###### Comparative material.

*Bisetifercephalotus* • 1 ♀ (TNU), Russia, Krasnodar Territory, Caucasus Nature Reserve, 20 km SSW of Psebay, 1 km SW of the cordon of Tshernoretshie, Urushten River bank, forest, 10.VI.2017, A.V. Ponomarev leg.

###### Distribution.

The Crimean-Caucasian disjunctive: Krasnodar Territory and the Republic of Adygea, Russia. The species has been recorded from Crimea for the first time, with the Crimean Mountains being currently the westernmost part of its range (Tanasevitch et al. 2015; present data).

###### Records from the Crimean caves.

Map (Fig. 17B – blue circle). Kizil-Koba Cave on western slope of the Dolgorukovskaya Yaila (present data).

###### Ecology.

**Figure 6.**
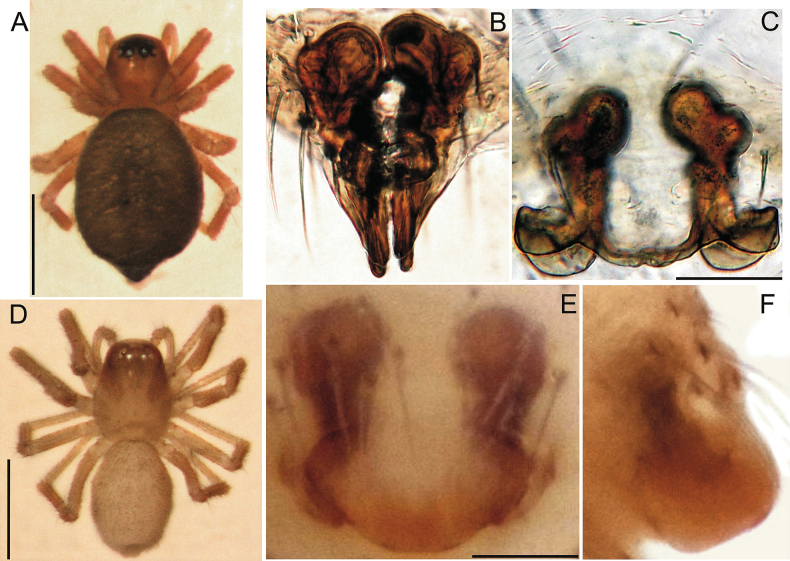
Females of *Bisetifercephalotus* from the Caucasus Nature Reserve (**A, B**), *B.gruzin* from Kizil-Koba Cave (**C, D**), paratype of *B.tactus* sp. nov. from Tshernoretshenskaya Cave (**E, F**). **A, D** habitus, dorsal view **B, C, E** epigyne, ventral view **F** epigyne, lateral view. Scale bars: 0.5 mm (**A, D**); 0.05 mm (**B, C, E, F**).

In the Caucasus, *B.gruzin* inhabits humid microbiotopes (Tanasevitch 1987; Tanasevitch et al. 2015). In Crimea, it was found in the upper floors of Kizil-Koba Cave, with no permanent water flow (Kovblyuk 2007; present data). The body of the Crimean specimens is depigmented, but the eyes are well developed (see Fig. 6D). Despite the well-studied araneofauna of Crimea, *B.gruzin* has never been reported from epigeic biotopes, whereas all our findings are from caves. On this basis, this species could be preliminary considered an eutroglophile.

###### Remarks.

In Crimea, two males of another congener, *B.cephalotus*, were collected earlier from Kizil-Koba Cave (Kovblyuk 2007); this material is currently stored by Valery A. Gnelitsa (Sumy, Ukraine). Since the earlier records of *B.cephalotus* and the newly collected specimens of *B.gruzin* come from the same cave, it could be suspected that they belong to the same species – *B.gruzin*.

In 2007, *B.gruzin* yet had not been described. This could have been the reason for erroneous identification, as *Bisetifer* species are better identified by the females (see Fig. 6B, C), while the males have a rather similar conformation of diagnostically important characters. Possible mistakes in the identification of *B.cephalotus* for Crimea were discussed by Tanasevitch et al. (2015), and their conclusion has been confirmed by present data.

##### ﻿ Bisetifer tactus sp. nov.

Taxon classificationAnimaliaAraneaeLinyphiidae

AF2939BE-8B72-5B6B-A73B-1C6DFC17B210

https://zoobank.org/80D9BAAF-5BF5-4C07-BB48-8CFC1147420F

**Figure 7.**
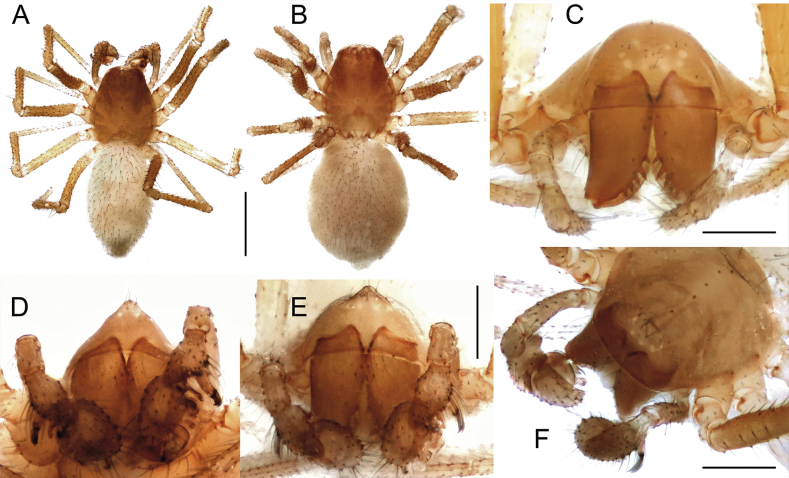
General appearance of male holotype and female paratype of *Bisetifertactus* sp. nov. from Tshernoretshenskaya Cave: **A, B** male and female habitus, dorsal **C** female prosoma, anteriorly **D, E** male prosoma, anteriorly in different aspects **F** male prosoma, dorsal. Scale bars: 0.5 mm (**A, B**); 0.2 mm (**C–F**).

**Figure 8.**
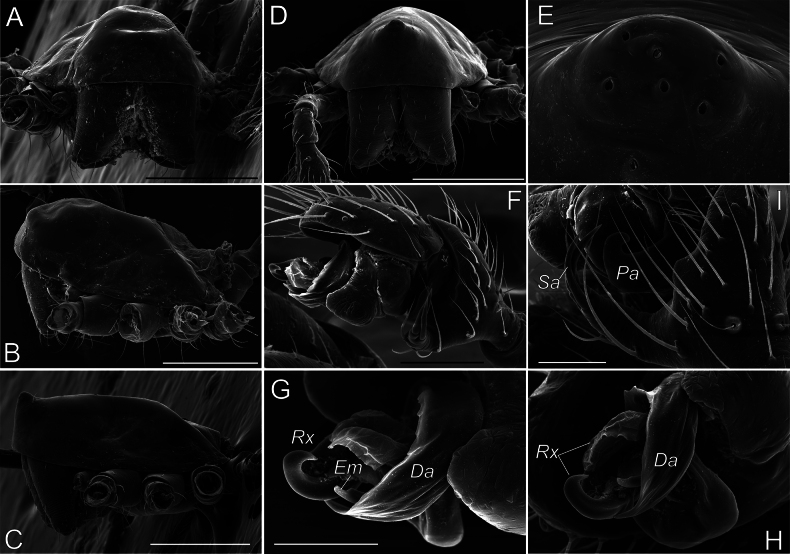
Details of female paratype and male holotype of *Bisetifertactus* sp. nov. from Tshernoretshenskaya Cave: **A, B** female prosoma, anterior and lateral views **C, D** male prosoma, lateral and anterior views **E** top of head part of male carapace, anterior view **F** male palp, retrolateral view **G, H** embolic division, ventro-retrolateral and ventro-apical views **I** fragment of male palpal tibia and paracymbium, posterior view. Abbreviations: *Da* – distal suprategular apophysis, *Em* – embolus, *Pa* – paracymbium, *Rx* – radix, *Sa* – setae at apex of palpal tibial apophysis. Scale bars: 0.3 mm (**A–D**); 0.1 mm (**F**); 0.05 mm (**G–I**).

**Figure 9.**
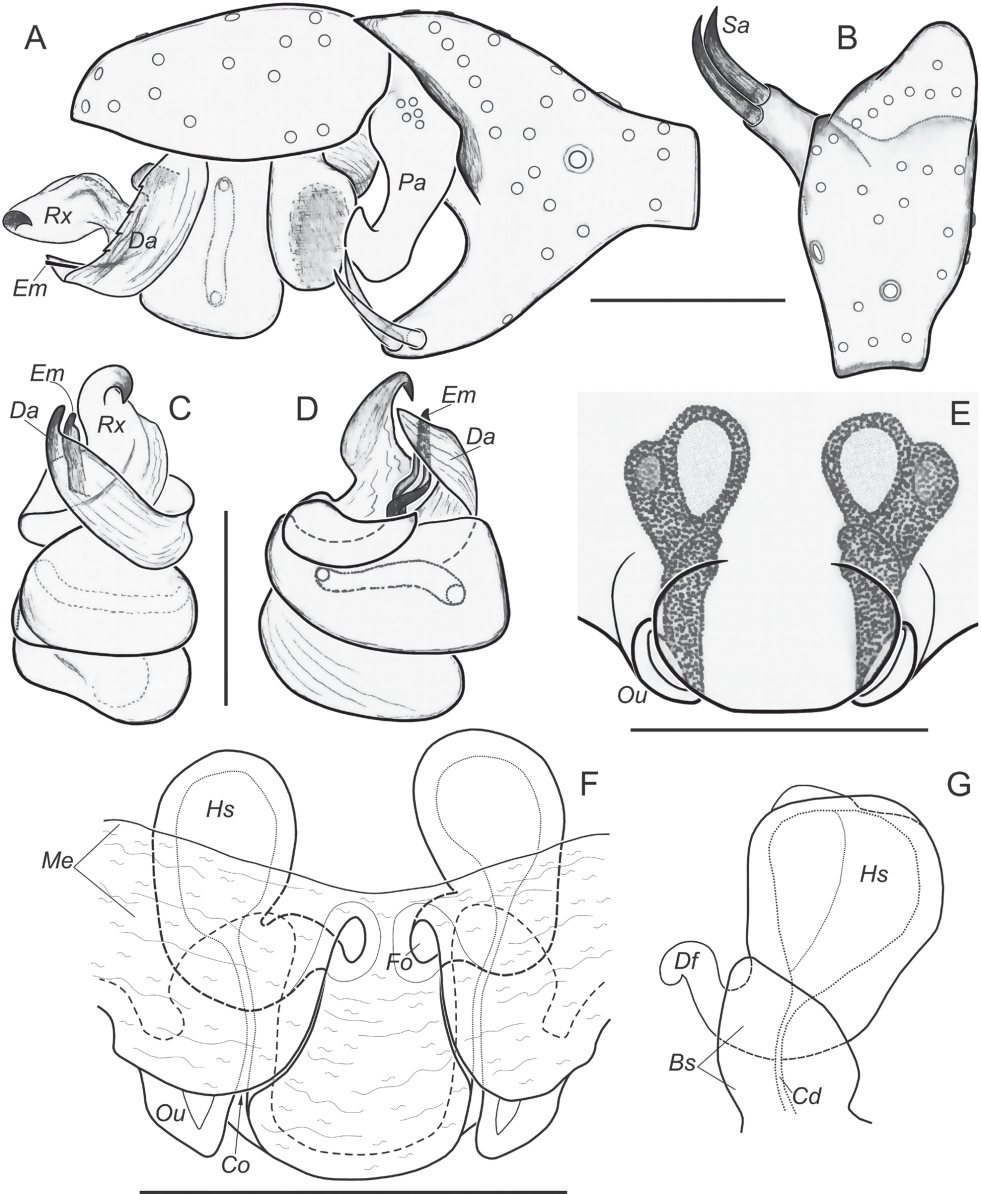
Male holotype and female paratype copulatory organs of *Bisetifertactus* sp. nov. from Tshernoretshenskaya Cave: **A** male palp, retrolateral view **B** male palpal tibia, dorsal view **C, D** bulbus, ventral and prolateral views **E, F** epigyne, ventral and dorsal views **G** spermatheca, ventral view. Abbreviations: *Bs* – base of spermatheca, *Cd* – copulatory duct, *Co* – copulatory opening, *Da* – distal suprategular apophysis, *Df* – fertilisation duct, *Em* – embolus, *Fo* – fertilisation opening, *Hs* – head of spermatheca, *Me* – membrane of spermatheca, *Ou* – outgrowths of epigyne, *Pa* – paracymbium, *Rx* – radix, *Sa* – setae at apex of palpal tibial apophysis. Scale bars: 0.1 mm (**A–F**); not scaled (**G**).

Figs 6E, F , 7 , 8 , 9

###### Type material.

***Holotype*** • ♂ (ZMMU Ta-8255), Crimea, nr Sevastopol, Tshernaya River canyon, Tshernoretshenskaya Cave, 3.III.2018, I.S. Turbanov leg. ***Paratypes*** • 3 ♀♀ (ZMMU Ta-8256), 5.V.2017 • 1 ♀ (TNU 10235), 4.V.2018, same cave and collector as for a holotype.

###### Diagnosis.

*Bisetifertactus* sp. nov. has reduced eyes (Figs 7A–F, 8A–D) (vs other congeners, *B.cephalotus* and *B.gruzin*, have well developed eyes, see Tanasevitch et al. 2015: figs 1–6). Additionally, *B.tactus* sp. nov. differs from its congeners in having: 1) the embolus hidden between radix and distal suprategular apophysis (Figs 8G, H, 9A, C, D) (vs not hidden, well visible, see Tanasevitch et al. 2015: figs 7, 19); 2) the hook-shaped and pointed apical part of radix (Figs 8G, 9C) (vs conical in *B.cephalotus* and flat in *B.gruzin*, see Tanasevitch et al. 2015: figs 9, 14, 23, 28–29); 3) the distal suprategular apophysis without a complicated arrangement of apophyses, with barbs on its edge (Figs 8G, 9A, C, D) (vs with apophyses, without barbs, see Tanasevitch et al. 2015: figs 7, 19); 4) the oval posterior edge of epigyne (Figs 6E, 9E) (vs with nipple-shaped outgrowths in *B.cephalotus*, with bow-shaped outgrowths in *B.gruzin*, see Fig. 6B, C and Tanasevitch et al. 2015: figs 17, 30).

###### Description.

**Male.** Total length 1.5. Carapace 0.63 long, 0.5 wide, pale brown; modified as in Figs 7D, E, 8C, D: head part conical, with setae. Eyes reduced, almost completely disappeared (head part with small pale spots, visible under light microscope; no lens visible under SEM). Chelicerae 0.31, brownish, transverse shallow cuticular grooves throughout the basal segment. Legs pale brown, chaetotaxy 2.2.1.1, metatarsi I–IV spineless, metatarsi IV without trichobothrium, TmI 0.35, leg I 2.47 long (0.69+0.18+0.63+0.52+0.45), leg IV 2.53 long (0.71+0.17+0.69+0.54+0.42). Palp as in Figs 8F–I, 9A–D: tibia with a ventro-retrolateral apophysis and two large setae on its tip, distally setae poorly serrate; paracymbium L-shaped; distal suprategular apophysis – flat, curved, and pointed, with barbs on its anterior edge; embolus small, situated in a cavity between distal suprategular apophysis and radix; apical part of radix hook-shaped and pointed distally, well-sclerotised process, retrolaterally with membrane. Abdomen pale grey.

**Female.** Total length 1.58. Carapace 0.77 long, 0.59 wide; unmodified. Eyes reduced, almost completely disappeared (head part with small pale spots, clearly visible under light microscope; a few poorly developed lenses visible under SEM). Chelicerae 0.36, transverse shallow cuticular grooves throughout the basal segment. TmI 0.44. Leg I 2.64 long (0.73+0.21+0.7+0.54+0.46), leg IV 2.78 long (0.8+0.2+0.77+0.59+0.42). Body colouration and spination as in the male. Epigyne as in Figs 6E, F, 9E–G: epigynal plate oval, with lateral outgrowths in which copulatory ducts open; spermathecae consists of two parts: base with copulatory duct and head with receptacle and fertilisation duct; cavity of receptacle subdivided on ventral and dorsal parts.

###### Variation.

Females (*n* = 3): carapace width 0.53–0.59; femur I length 0.69–0.73.

###### Distribution and records from the Crimean caves.

Map (Fig. 17B – purple circle). Only known from the type locality: Tshernoretshenskaya Cave, nr Sevastopol.

###### Ecology.

The species has troglomorphic characteristics related to the subterranean habitat, such as the pale body and reduced eyes. Based on the morphological features and the fact that this species is known only from caves, it can be considered a troglobiont.

###### Etymology.

From the Latin *tactus*, meaning touch, due to the fact that this species has the strongly reduced eyes and its life style as a true troglobiont relies on tactile sensations.

#### ﻿Genus *Caviphantes* Oi, 1960

##### ﻿ Caviphantes dobrogicus

Taxon classificationAnimaliaAraneaeLinyphiidae

(Dumitrescu & Miller, 1962)

1974A443-2609-5F72-B646-188D884B9FF3

Figs 1C , 10

Caviphantes dobrogicus (Dumitrescu & Miller, 1962): Turbanov et al. 2021: 180–181, 183–184, figs 2, 3.

###### Material examined.

• 5 ♀♀ (TNU-10234), Crimea, Belogorsk Distr., nr Zuya Vil., Tavrida Cave, 29.IX.2018, I.S. Turbanov leg.

###### Distribution.

West and Central Palaearctic nemoral-subtropical: Bulgaria, Romania, Ukraine, the southern part of European Russia, Georgia, Azerbaijan, Kyrgyzstan (Nentwig et al. 2024).

###### Records from the Crimean caves.

Map (Fig. 17B – green circle). Tavrida Cave nr Zuya Vil. (Turbanov et al. 2021).

###### Ecology.

The species has been considered a troglophile (Mammola et al. 2018: table S1). In addition to caves, it inhabits sandy steppes, sea coasts and agrocenoses (Polchaninova and Prokopenko 2013). Since this species is a eurybiont that can enter caves, Turbanov et al. (2021) characterised it as a subtroglophile. Despite the Crimean araneofauna is well studied, *C.dobrogicus* has never been reported from epigeic biotopes. Based on the fact that in Crimea, the species is likely to be permanently associated with caves, it could be considered an eutroglophile.

###### Remarks.

**Figure 10.**
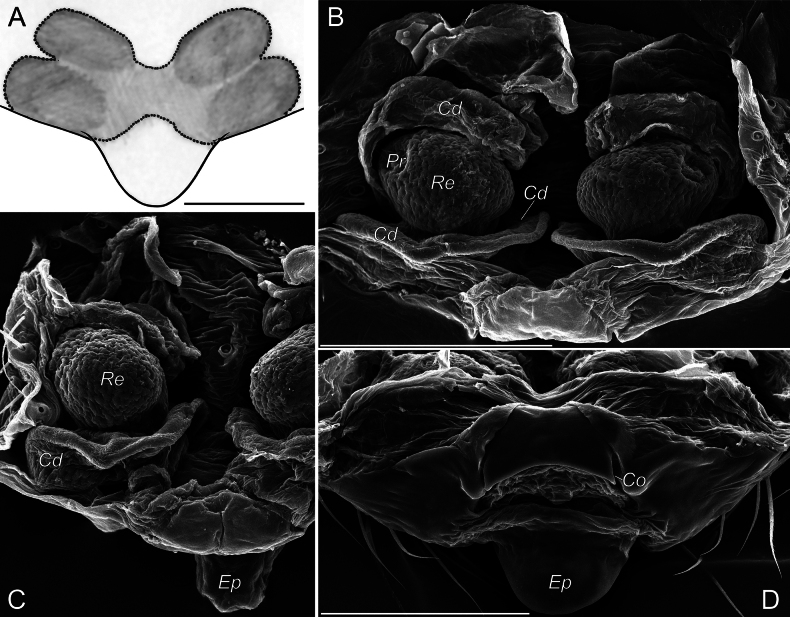
Epigynes of *Caviphantesdobrogicus* from Tavrida Cave: **A** ventral view **B, C** dorsal view **D** posterior view. Abbreviations: *Cd* – copulatory duct, *Co* – copulatory opening, *Ep* – epigynal plate, *Pr* – pore of receptacle, *Re* – receptacle. Scale bars: 0.1 mm.

Only a few line drawings and digital photos of important diagnostic features of this tiny spider have been published (see WSC 2024). We present SEM micrographs of the vulva, which add to the understanding of the structure of its membranous parts (Fig. 10B–D). It is somewhat different from what can be seen under light microscope (Turbanov et al. 2021) and was provided by the original description (Dumitrescu and Miller 1962). The structures termed as the copulatory ducts are poorly sclerotised and in fact wide, but not like a twisted system of narrow ducts.

#### ﻿Genus *Lepthyphantes* Menge, 1866

##### ﻿ Lepthyphantes leprosus

Taxon classificationAnimaliaAraneaeLinyphiidae

(Ohlert, 1865)

0D4CC272-75D7-56DD-8057-C2A21F3DDF0D

Lepthyphantes leprosus (Ohlert, 1865): Evtushenko 2004: 66–68; Kovblyuk 2014: 44; Zagorodniuk and Vargovitsh 2004: 207; Turbanov et al. 2016b: 1283; Samokhin et al. 2019: 247.

###### Material examined.

• 1 ♂ (TNU 10236/2), Crimea, Bakhchisarai Distr., nr Khodzha-Sala Vil., steep southern slope of Baba-Dagh Plateau (= Mangup-Kale Gorodishche), entrance to Mangupskaya I (= MK-1) Cave, 11.VI.2018, I.S. Turbanov, A.A. Nadolny leg. • 3 ♀♀ (TNU 10180/3), Crimea, Sevastopol, nr Balaklava, Aya Cape Mt. Range, Kala-Fatlar Mt., entrance to Gekkonovaya Cave, 9.X.2016, A.A. Nadolny leg. • 7 ♀♀ (IT), Crimea, nr Sevastopol, Tshernaya River canyon, entrance to Tshernoretshenskaya Cave, 3.VI.2021, I.S. Turbanov, A.A. Nadolny leg. • 4 ♂♂ 5 ♀♀ (TNU 10257/1) • 1 ♀ (IT), Crimea, nr Sevastopol, northwestern slope of Ai-Petri Yaila, Baidarskaya Valley, nr Rodnikovskoye Vil., entrance to Skelskaya Cave, 29.IX.2020, A.A. Nadolny, I.S. Turbanov A.A. Turbanova leg.

###### Distribution.

Circum-Holarctic polyzonal (Kovblyuk and Kastrygina 2015; Nentwig et al. 2024).

###### Records from the Crimean caves.

Map (Fig. 17B – red circle). Recorded in caves from the western and central parts of the Crimean Mountains: in unnamed cave near the city of Bakhchisarai, Mangupskaya I, Gekkonovaya, Tshernoretshenskaya, Skelskaya, and Kizil-Koba (Evtushenko 2004; present data).

###### Ecology.

A troglophile and northward, above the 55^th^ parallel, exclusively as a synanthropic species (Kovblyuk and Kastrygina 2015; Mammola et al. 2018; Nentwig et al. 2024). In Crimea, the species lives in the mountainous regions, except for its upper parts – mountain meadows and yaila steppes (Kovblyuk and Kastrygina 2015), and is confined to cave entrances; in our opinion, it is a subtroglophile.

#### ﻿Genus *Megalepthyphantes* Wunderlich, 1994

##### ﻿ Megalepthyphantes nebulosus

Taxon classificationAnimaliaAraneaeLinyphiidae

(Sundevall, 1830)

2E38735C-CDDB-599B-A998-C889DA6E1078

###### Material examined.

• 1 ♂ (TNU 10180/2), Crimea, Sevastopol, nr Balaklava, Aya Cape Mt. Range, Kala-Fatlar Mt., Gekkonovaya Cave, 9.X.2016, A.A. Nadolny leg.

###### Distribution.

Holarctic polyzonal (Kovblyuk and Kastrygina 2015; Nentwig et al. 2024).

###### Records from the Crimean caves.

Map (Fig. 17B – pale blue circle). Gekkonovaya Cave of Aya Cape Mt. Range (present data).

###### Ecology.

Above the 55^th^ parallel northwards it is an exclusively synanthropic species (Kovblyuk and Kastrygina 2015; Nentwig et al. 2024), but southwards it can be found in natural biotopes – under stones and in rock crevices (Tyshchenko 1971). In Crimea, *M.nebulosus* is rare, recorded in Sevastopol and Feodosia (Kovblyuk and Kastrygina 2015), and only once in the subterranean biotopes (present data). Also, this species was recorded as a troglophile in the Kristalnaya Cave in Ternopol region, Ukraine (Evtushenko 2004; Zagorodniuk and Vargovitsh 2004). In the Crimean caves, the ecological confinement of *M.nebulosus* is not entirely clear, it is probably a subtroglophile.

##### ﻿ Megalepthyphantes pseudocollinus

Taxon classificationAnimaliaAraneaeLinyphiidae

Saaristo, 1997

87107F5C-CE54-5FA1-A3EE-59607B22745A

Fig. 1D

###### Material examined.

• 2 ♀♀ (TNU 10257/2), Crimea, nr Sevastopol, NW slope of Ai-Petri Yaila, Baidarskaya Valley, nr Rodnikovskoye Vil., entrance to Skelskaya Cave, 29.IX.2020, A.A. Nadolny leg.

###### Distribution.

West and Central Palaearctic nemoral: from Central Europe to West Siberia and from Finland to Iran (Kovblyuk and Kastrygina 2015; Nentwig et al. 2024).

###### Records from the Crimean caves.

Map (Fig. 17B – white circle). Skelskaya Cave in Baidarskaya Valley (present data).

###### Ecology.

In Crimea, *M.pseudocollinus* was reported from the Karadag Nature Reserve in Feodosia District (Kovblyuk and Kastrygina 2015). Previously, this species was referred to as a trogloxene in Kungurskaya Ledyanaya (= Kungur Ice) Cave in Perm Oblast of Russia (Pankov et al. 2009). Since in Crimea the species was found at the cave entrance, it is likely to be a trogloxene species.

#### ﻿Genus *Palliduphantes* Saaristo & Tanasevitch, 2001

##### ﻿ Palliduphantes khobarum

Taxon classificationAnimaliaAraneaeLinyphiidae

(Charitonov, 1947)

9038DAF3-A728-56A9-B6AF-5BA26650482A

Fig. 1E

Lephthyphantes [sic!] khobarum Charitonov, 1947: Charitonov 1947a: 45–47, 49, 52–53, figs 1–3; Charitonov 1947b: 1.
Lepthyphantes khobarum Charitonov, 1947: Birstein 1963: 128; Tyshchenko 1971: 23; Brignoli 1980: 190; Tanasevitch 1987: 314; Mikhailov 1997: 73; Kovblyuk 2002: 104; Amelichev et al. 2004: 133, 140; Evtushenko 2004: 66–68; Kovblyuk 2004c: 251, 253–254, 256; Zagorodniuk and Vargovitsh 2004: 207.
Palliduphantes khobarum (Charitonov, 1947): Kovblyuk 2004a: 230; Mikhailov 2013: 78; Kovblyuk 2014: 44; Kovblyuk and Kastrygina 2015: 31–32; Turbanov et al. 2016b: 1283–1284; Samokhin and Turbanov 2019: 230.
Lephthyphantes [sic!] monticola Kulcz.: Novikov 1912: 104; Lebedinsky 1914: 127; Mokrzecki 1914: 97.
Lepthyphantes monticola (Kulczynski, 1881): Charitonov 1932: 75; Charitonov 1939: 197; Mikhailov 1997: 74.
Anguliphantes monticola (Kulczynski, 1881): Kovblyuk 2004a: 226; Mikhailov 2013: 42; Kovblyuk 2014: 44; Turbanov et al. 2016b: 1283.

###### Material examined.

• 1 ♂ (TNU 10264), Crimea, Sevastopol, nr Oboronnoye Vil., Ayu-Kaya Mt., Kay-Kobasy Cave, 6.IV.2019, S.V. Arefiev leg. • 1 ♀ (TNU 10231/2), Crimea, nr Sevastopol, Tshernaya River canyon, Tshernoretshenskaya Cave, 3.III.2018, A.A. Nadolny leg. • 2 ♀♀ (TNU 10262), same cave, 15.I.2020, I.S. Turbanov leg. • 1 ♀ (TNU 10287/2), same cave, 3.VI.2021, I.S. Turbanov, A.A. Nadolny leg. • 1 ♀ (TNU 10224), Crimea, nr Sevastopol, northwestern slope of Ai-Petri Yaila, Baidarskaya Valley, nr Pavlovka Vil., Baidar-Tshokrak Cave, 28.V.2015, I.S. Turbanov leg. • 1 ♀ (TNU 10183), Crimea, nr Sevastopol, northwestern slope of Ai-Petri Yaila, Baidarskaya Valley, nr Rodnikovskoye Vil., Skelskaya Cave, 3.III.2015, I.S. Turbanov leg. • 1 ♂ 5 ♀♀ (IT), same cave, 29.IX.2020, I.S. Turbanov, A.A. Turbanova leg. • 1 ♀ (TNU 10232), same cave, 4.III.2018, I.S. Turbanov, A.A. Turbanova leg. • 2 ♀♀ (TNU 10238/1), same cave, 25.IX.2018, I.S. Turbanov, A.A. Turbanova leg. • 1 ♀ (IT), Crimea, nr Sevastopol, western part of Ai-Petri Yaila, Karadagh Forest, Zemlyanitshnaya Cave, 18.VI.2011, I.S. Turbanov leg. • 2 ♀♀ (TNU 10199/1), Crimea, nr Sevastopol, western part of Ai-Petri Yaila, Karadagh Forest, Kristalnaya (= Maksimovitcha) Cave, 1.V.2013, I.S. Turbanov leg. • 1 ♂ (TNU 10183) • 1 ♂, 2 ♀♀ (IT), same cave, 6.X.2020, I.S. Turbanov leg.• 2 ♂♂ 4 ♀♀ (TNU 10263), Crimea, Bakhchisarai Distr., northern part of Ai-Petri Yaila, nr Maly Babulghan, Villyaburunskaya Cave, 6.V.2015, I.S. Turbanov leg. • 1 ♂ (TNU 10222), Crimea, Bakhchisarai Distr., northeastern slope of Ai-Petri Yaila, nr Bash-Dere, Avantyura Cave, 15.XI.2014, I.S. Turbanov leg. • 1 ♂ 1 ♀ (TNU 10186), Crimea, Simferopol Distr., central part of Tshatyr-Dagh Yaila, Vyalovsky Forest, Paskhalnaya Cave, 12.II.2015, I.S. Turbanov leg. • 1 ♀ (TNU 10195/3), Crimea, Simferopol Distr., northern part of Tshatyr-Dagh Yaila, Binbash-Koba Cave, 12.II.2015, I.S. Turbanov leg. • 1 ♀ (TNU 10188), Crimea, Simferopol Distr., nr Perevalnoye Vil., western slope of the Dolgorukovskaya Yaila, Kizil-Koba (= Krasnaya) Cave, 9.XI.2014, A.A. Nadolny leg. • 1 ♀ (TNU 10225), Crimea, Simferopol Distr., central pаrt of the Dolgorukovskaya Yaila, Sliyanie Cave, 23.VII.2017, I.S. Turbanov leg. • 1 ♀ (TNU 10182), Crimea, Simferopol Distr., eastern part of Dolgorukovskaya Yaila, Vostotshny Potok Cave, 22.II.2014, I.S. Turbanov leg. • 2 ♀♀ (TNU 10198), Crimea, Simferopol Distr., eastern part of Dolgorukovskaya Yaila, Partizanskaya Cave, 5.IV.2014, I.S. Turbanov leg. • 5 ♂♂ 8 ♀♀ (TNU 10192), Crimea, nr Alushta, south-eastern slope of Karabi Yaila, Tuakskaya (= Ful-Koba) Cave, 8.V.2012, I.S. Turbanov leg.

###### Distribution.

East Mediterranean: Greece, Turkey, Ukraine, the south part of European Russia, Georgia, Azerbaijan, Iran. Crimea lies at the northernmost limit of the species range (Kovblyuk and Kastrygina 2015; Nentwig et al. 2024).

###### Records from the Crimean caves.

Map (Fig. 17B – orange circle). Known from the Crimean caves of Sevastopol in the west to Karabi Yaila in the east: Kay-Kobasy, Tshernoretshenskaya, Baidar-Tshokrak, Skelskaya, Zemlyanitshnaya, Kristalnaya, Villyaburunskaya, Ayu-Teshik, Avantyura, Paskhalnaya, Binbash-Koba, Kizil-Koba, Sliyanie, Vostotshny Potok, Partizanskaya, Tisovaya, and Tuakskaya (type locality of *P.khobarum*) (Novikov 1912; Lebedinsky 1914; Charitonov 1947a; present data).

###### Ecology.

A troglophile (Mammola et al. 2018). *Palliduphanteskhobarum* is found everywhere in the mountains and on the southern coast of Crimea (Kovblyuk and Kastrygina 2015). One of the most widespread and common spiders in the Crimean caves, apparently capable of maintaining permanent populations in subterranean biotopes; can be classified as an eutroglophile.

###### Remarks.

*Palliduphanteskhobarum* was described based on the spider collection by Lebedinsky (1914). According to Charitonov (1947a, b), the reports of *Lepthyphantesmonticola* (now, *Anguliphantesmonticola*) for the Crimean caves (Novikov 1912; Lebedinsky 1914; Mokrzecki 1914; Charitonov 1932, 1939) in fact belong to *P.khobarum*. Yet, a number of researchers have erroneously mentioned *A.monticola* as occurring in Crimea (Mikhailov 1997, 2013; Kovblyuk 2004a, 2014). Later, *A.monticola* was excluded from the list of species of Crimea (Kovblyuk and Kastrygina 2015: 32).

#### ﻿Genus *Tenuiphantes* Saaristo & Tanasevitch, 1996

##### ﻿? Tenuiphantes zimmermanni

Taxon classificationAnimaliaAraneaeLinyphiidae

(Bertkau, 1890)

1DAB9695-5CE1-56FC-9724-6F5BFB6C6045

? Lepthyphanteszimmermanni Bertkau, 1890: Evtushenko 2004: 67–68; Zagorodniuk and Vargovitsh 2004: 207.
? Tenuiphanteszimmermanni (Bertkau, 1890): Mikhailov 2013: 93; Kovblyuk 2014: 44; Kovblyuk and Kastrygina 2015: 34.

###### Distribution.

West Palearctic polyzonal: from Portugal to the European part of Russia and from Scandinavia to Turkey (Kovblyuk and Kastrygina 2015; Nentwig et al. 2024).

###### Records from the Crimean caves.

Map (Fig. 17B – pink circle). Troizkogo (= Kharkovskaya, ZUG) Cave in E part of Ai-Petri Yaila and Tisovaya Cave in central part of Karabi Yaila (Evtushenko 2004).

###### Ecology.

A troglophile (Mammola et al. 2018). Mentioned from Crimea as a probable trogloxene (Evtushenko 2004).

###### Remarks.

According to some publications (Mikhailov 2013; Kovblyuk 2014; Kovblyuk and Kastrygina 2015), the record of *T.zimmermanni* from Crimea is questionable and not supported by the collected material.

#### ﻿Genus *Troglohyphantes* Joseph, 1881

##### ﻿ Troglohyphantes exspectatus sp. nov.

Taxon classificationAnimaliaAraneaeLinyphiidae

2E726BCE-8675-5DF2-A70F-494A399CD036

https://zoobank.org/1C70B3DF-8B98-4916-A04B-241A071463FB

**Figure 11.**
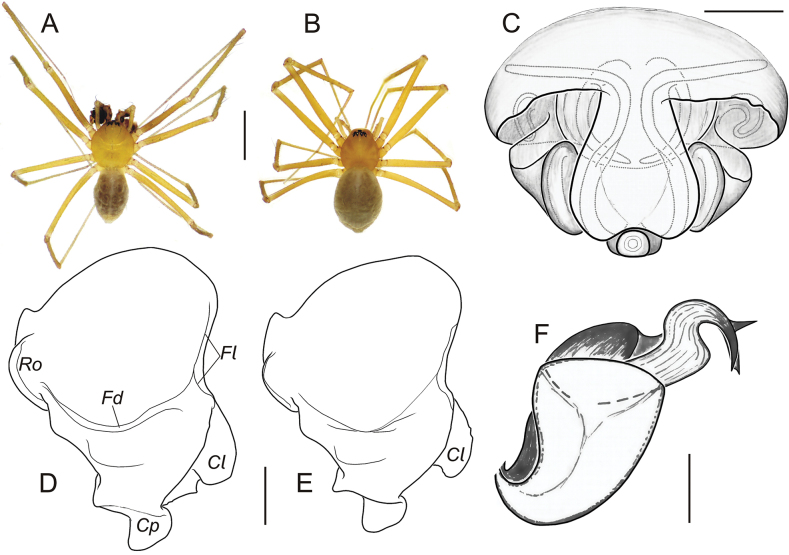
Habitus and copulatory organs of *Troglohyphantesexspectatus* sp. nov. from Druzhba Cave (paratypes): **A, B** male and female, dorsal view **C** epigyne, ventral view **D, E** cymbium, dorsal views **F** distal part of radix and lamella. Abbreviations: *Cl* – prolateral process, *Cp* – posterior process, *Fd* – dorsal furrow, *Fl* – prolateral furrow, *Ro* – rounded deflection. Scale bars: 1.0 mm (**A, B**); 0.1 mm (**C–F**).

**Figure 12.**
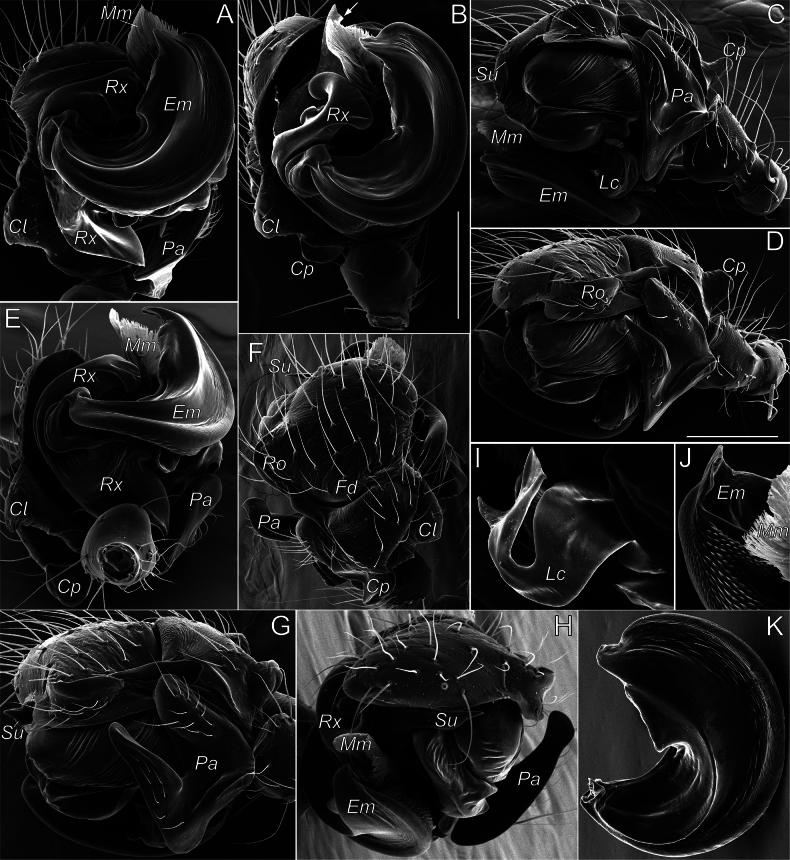
Male palps of *Troglohyphantesexspectatus* sp. nov. from Druzhba Cave (paratype): **A, B** palps, ventral in different aspects (arrow in B indicates ridge of *Su*) **C, D** palp, retrolateral views **E** palp, posterior view **F** palp, dorsal view **G** palp with focus on paracymbium, posterio-retrolateral view **H** palp, anterior view **I** lamella, posterior view **J** tip of embolus, dorsal view **K** embolus, ventral view. Abbreviations: *Cl* – prolateral cymbial process, *Cp* – posterior cymbial process, *Em* – embolus, *Fd* – dorsal furrow of cymbium, *Lc* – lamella, *Mm* – median membrane, *Pa* – paracymbium, *Ro* – rounded deflection of cymbium; *Rx* – radix, *Su* – suprategular apophysis. Scale bars: 0.2 mm (**B, D**); not scaled (**A, C, E–K**).

**Figure 13.**
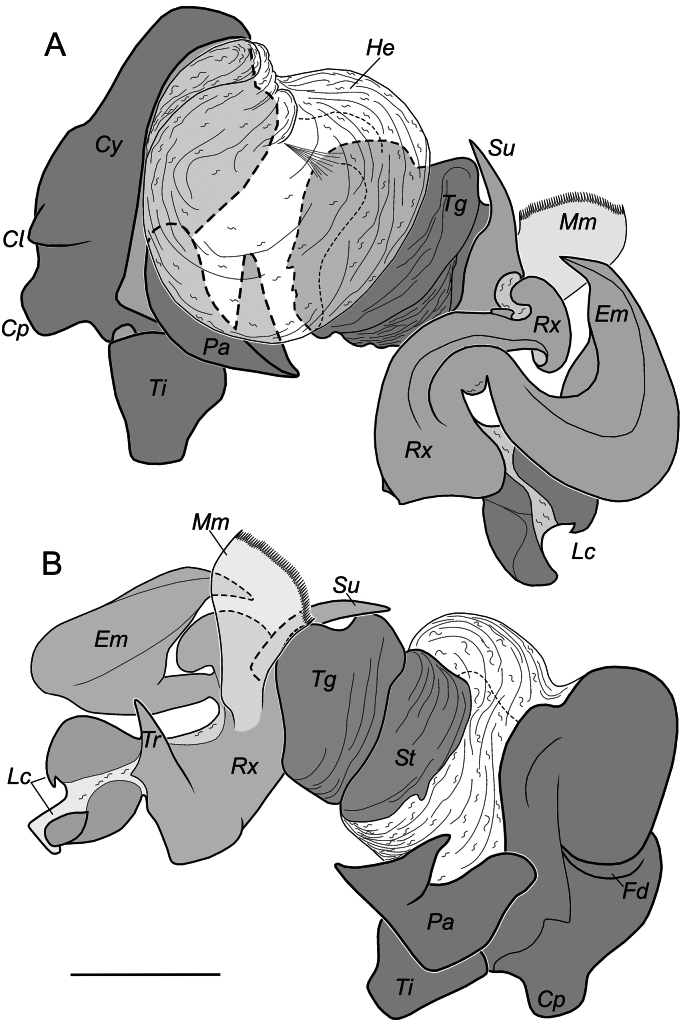
Palp with expanded bulbus of *Troglohyphantesexspectatus* sp. nov. from Druzhba Cave (paratype): **A, B** cymbium in prolateral and retrolateral positions. Abbreviations: *Cy* – cymbium, *Cl* – prolateral cymbial process, *Cp* – posterior cymbial process, *Em* – embolus, *Fd* – dorsal furrow of cymbium, *He* – haemathodoha, *Lc* – lamella characteristica, *Mm* – median membrane, *Pa* – paracymbium, *Rx* – radix, *Su* – suprategular apophysis, *St* – subtegulum, *Tg* – tegulum, *Ti* – tibia, *Tr* – terminal apophysis. Scale bar: 0.2 mm.

**Figure 14.**
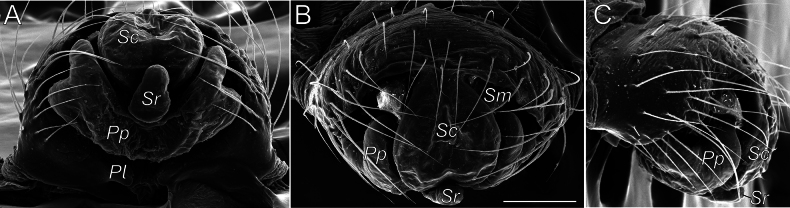
Epigyne of *Troglohyphantesexspectatus* sp. nov. from Druzhba Cave (paratype): **A–C** posterior, ventral, and lateral views. Abbreviations: *Pl* – posterior lobes, *Pp* – posterior plate, *Sc* – scape, *Sm* – median part of scape, *Sr* – stretcher. Scale bar: 0.1 mm.

Figs 1F , 11 , 12 , 13 , 14

###### Type material.

***Holotype*** • ♂ (ZMMU Ta-8257), Crimea, nr Sevastopol, SW part of Ai-Petri Yaila, Mortsheka Mt., Druzhba Cave, 3.X.2020, I.S. Turbanov leg. ***Paratypes*** • 1 ♀ (ZMMU Ta-8258), 4.V.2015 • 8 ♀♀ (ZMMU Ta-8259), 22.IX.2018 • 1 ♂ 1 ♀ (TNU 10289), 3.X.2020, same cave and collector as for the holotype.

###### Other material examined.

• 3 juv. ♀♀ 4 subad. ♂♂ (TNU 10233), 22.IX.2018 • 9 ♀♀ 4 juv. (IT), 3.X.2020, same cave and collector as for the holotype.

###### Diagnosis.

*Troglohyphantesexspectatus* sp. nov. is most similar to the Bulgarian endemic *T.drenskii* Deltshev, 1973 (the *salax* group sensu Deeleman-Reinhold 1978). Two species can be easily distinguished by the eyes (in *T.exspectatus* sp. nov. well developed, with black pigmentation around, see Fig. 11A, B vs strongly reduced, without pigmentation in *T.drenskii*, see Deltshev 1973: fig. 1) and the clypeus (in *T.exspectatus* sp. nov. without modifications vs concave, with modification in *T.drenskii*, see Deltshev 1973: fig. 1). Structures of male palps are almost identical in both species and differ in details of the cymbium (cf. Figs 11D, E, 12C, D and Deltshev 1973: figs 2, 4). Epigynes differ in the shape of scape: ventral max/min width ratio in *T.exspectatus* sp. nov. 1.9 and in *T.drenskii* 2.9 (cf. Fig. 11C and Deltshev 1973: fig. 7).

###### Description.

**Male** (paratype). Total length 2.12. Carapace 0.98 long, 0.88 wide, yellow. Palps, chelicerae, and legs yellow. Basal chelicerae segment 0.52 long. Leg I length: femur 1.9, patella 0.3, tibia 2.18, metatarsus 2.15, tarsus 1.18, TLL 7.71. Leg II length: femur 1.85, patella 0.3, tibia 2.0, metatarsus 1.92, tarsus 1.05, TLL 7.12. Leg III length: femur 1.55, patella 0.28, tibia 1.48, metatarsus 1.5, tarsus 0.8, TLL 5.61. Leg IV length: femur 1.85, patella 0.28, tibia 1.9, metatarsus 1.88, tarsus 1.0, TLL 6.91. Leg I spination: femur one dorsal and one prolateral spine; tibia two dorsal, two prolateral and two retrolateral spines; metatarsus one dorsal spine. Leg II spination: femur one dorsal spine; tibia two dorsal and one retrolateral spine; metatarsus one dorsal spine. Leg III spination: femur one dorsal spine; tibia two dorsal spines; metatarsus one dorsal spine. Leg IV spination: femur no spine; tibia one dorsal spine; metatarsus no spine. Metatarsi IV without trichobothrium. TmI 0.14. Palp as in Figs 11D, E, 12A–K, 13A, B: cymbium has two processes (prolateral and posterior), two furrows (dorsal and prolateral), rounded deflection in anterior-retrolateral edge, with hollows and ridges in retrolaleral part; paracymbium with two shallow furrows closely situated to each other; suprategular apophysis with a ridge; F-shaped proximal part of radix (two lobes and one small apophysis); median membrane joined with the proximal part of radix dorsally; the distal part of radix with flat pointed terminal apophysis; lamella characteristica with two sclerotised branches, clearly distinct when palp is expanded; embolus with a serrate area dorsally and a keel on prolateral side; cymbium length/width ratio 1.3 (same ratio with and without apophyses). Eyes normal. Abdomen grey.

**Female.** Total length 1.92. Carapace 0.88 long, 0.78 wide. Basal chelicerae segment 0.6 long. Leg I length: femur 1.68, patella 0.29, tibia 1.82, metatarsus 1.64, tarsus 0.98, TLL 6.41. Leg II length: femur 1.6, patella 0.29, tibia 1.66, metatarsus 1.51, tarsus 0.89, TLL 5.95. Leg III length: femur 1.35, patella 0.25, tibia 1.2, metatarsus 1.18, tarsus 0.68, TLL 4.66. Leg IV length: femur 1.6, patella 0.25, tibia 1.56, metatarsus 1.46, tarsus 0.81, TLL 5.68. Leg I spination: femur one dorsal and one prolateral spine; tibia one dorsal, one prolateral, one retrolateral, and four ventral spines; metatarsus one dorsal spine. Leg II spination: femur one dorsal spine; tibia two dorsal, one retrolateral and two ventral spines; metatarsus two dorsal spines. Leg II spination: femur one dorsal spine; tibia two dorsal spines; metatarsus two dorsal spines. Leg IV spination: femur no spine; tibia two dorsal spines; metatarsus no spine. Metatarsi IV without trichobothrium. TmI 0.15. Epigyne as in Figs 11C, 14A–C: in ventral view scape resembling a shape of water drop with truncated anterior side; posterior plate in caudal view looks bifurcated, each branch with two rounded protrusions; posteriorly sides of epigyne folded and terminate with posterior lobes, directed towards each other. Body colouration as in male.

###### Remarks.

The embolic and radix structure of the new species is similar to that of *T.adjaricus* Tanasevitch, 1987, *T.deelemanae* Tanasevitch, 1987, *T.lucifuga* (Simon, 1884), and other related species from the *orpheus* group (sensu Deeleman-Reinhold 1978 and Isaia et al. 2017), but can be distinguished by the pear-shape cymbium (in dorsal view), with two small apophyses in its proximal part (vs in *T.lucifuga* complex and *T.adjaricus* one or three apophyses, usually prolateral apophysis is large; in *T.deelemanae* with large prolateral apophysis, whose length is equal to width of middle part of cymbium). The shape of the lamella in the new species differs in detail from that of all the congeners. Some similarity can be found in new species and *T.cyrnaeus* Isaia, 2023 from the *salax* group (Isaia et al. 2023: fig. 2D). Both have S-shaped lamellae, but in *T.exspectatus* sp. nov. the end of the lamella is bifurcated with one branch pointed and the other flatted (Figs 11F, 12I). The epigyne of the new species is similar to those of *T.deelemanae* and *T.konradi* Brignoli, 1975 (see Isaia et al. 2011). Females of these species are distinguishable by the angle between lateral side of scape and edge of epigyne (in a new species sides are perpendicular to each other, with angle between side of scape and edge of epigyne ~ 70° vs subparallel in *T.deelemanae*) and the eye development (well developed in the new species, but reduced in *T.konradi*). Also, *T.exspectatus* sp. nov. has the epigyne similar to that of some species of the *salax* group (sensu Deeleman-Reinhold 1978; *T.strandi* Absolon & Kratochvil, 1932, *T.fallax* Deeleman-Reinhold, 1978, *T.lesserti* Kratochvil, 1935 – all of them have reduced eyes) and the embolus similar to those of the members of the *polyophthalmus* group (*T.inermis* Deeleman-Reinhold, 1978 is distinguishable by the shapes of the lamella and cymbium).

###### Variation.

Males (*n* = 2): carapace width 0.83–0.88; femur I length 1.81–1.9. Females (n = 9): carapace width 0.77–0.85; femur I length 1.67–1.9.

###### Distribution and records from the Crimean caves.

Map (Fig. 17B – yellow circle). Only known from the type locality: Druzhba Cave on Mortsheka Mt. in the SW part of Ai-Petri Yaila.

###### Ecology.

Given that *T.exspectatus* sp. nov. has the well-developed eyes but permanently occurs and reproduces in Druzhba Cave (we have recorded individuals at all developmental stages), it can be classified as an eutroglophile.

###### Etymology.

From the Latin *exspectatus*, meaning expected, due to the fact that we have not come across adult males of this species for a long time, but only females, subadult males, and juveniles.

### ﻿Family Lycosidae Sundevall, 1833

#### ﻿Genus *Alopecosa* Simon, 1885

##### ﻿ Alopecosa farinosa

Taxon classificationAnimaliaAraneaeLycosidae

(Herman, 1879)

07BFCA93-AA7B-5A61-B6AD-3DD63F76650D

###### Material examined.

• 1 ♂ (TNU 10199/2), Crimea, nr Sevastopol, western part of Ai-Petri Yaila, Karadagh Forest, Kristalnaya (= Maksimovitcha) Cave, 1.V.2013, I.S. Turbanov leg.

###### Distribution.

Transpalaearctic polyzonal (Kovblyuk and Kastrygina 2015; Nentwig et al. 2024).

###### Records from the Crimean caves.

Map (Fig. 17A – grey circle). Kristalnaya Cave on the western part of Ai-Petri Yaila.

###### Ecology.

*Alopecosafarinosa* is distributed throughout Crimea and is a common species in the mountains (Kovblyuk and Kastrygina 2015). This species has never been previously recorded in caves. Clearly, *A.farinosa* is an accidental species in caves, hence can be classified as a trogloxene species.

### ﻿Family Nesticidae Simon,1894

#### ﻿Genus *Aituaria* Esyunin & Efimik, 1998

##### ﻿ Aituaria borutzkyi

Taxon classificationAnimaliaAraneaeNesticidae

(Reimoser, 1930)

3EBFD638-2B7C-5B21-83CA-EA8B664E9604

Figs 2B , 15 , 16

###### Material examined.

• 3 ♀♀ (TNU 10226), Crimea, Bakhchisarai Distr., nr Khodzha-Sala Vil., steep southern slope of Baba-Dagh Plateau (= Mangup-Kale Gorodishche), Mangupskaya I (= MK-1) Cave, 28.IV.2017, I.S. Turbanov leg. • 10 ♀♀ (TNU 10237/1), same cave, 3.V.2018, I.S. Turbanov leg. • 3 ♀♀ (TNU 10266), same cave, 6–8.V.2017, O.L. Makarova, K.V. Makarov leg. • 1 ♂ (TNU 10236/1), same cave, 11.VI.2018, I.S. Turbanov, A.A. Nadolny leg. • 1 ♂ 1 ♀ (TNU 10273), same cave, 2.X.2020, I.S. Turbanov, A.A. Turbanova leg.

###### Comparative material.

• 1 ♂ (TNU 10274), southern part of Simferopol, indoors; 27.VI.2011; A.A. Nadolny leg.

###### Distribution.

Minor Asia (Turkey), the west Caucasus (Abkhazia) and Crimea (Nadolny and Kovblyuk 2007).

###### Records from the Crimean caves.

Map (Fig. 17B – black circle). Mangupskaya I Cave on a steep southern slope of Baba-Dagh Plateau (present data).

###### Ecology.

A troglophile and synanthropic species (Nadolny and Kovblyuk 2007; Mammola et al. 2018; present data). In Crimea, *A.borutzkyi* has been found in anthropogenic biotopes of Simferopol and Fersmanovo Vil. (Nadolny and Kovblyuk 2007, present data). During the present study, it was found only in Mangupskaya I Cave on Baba-Dagh Plateau. On this plateau there was the city of Dori (= Doros), the capital of the medieval late Byzantine Orthodox Principality of Theodoro (13^th^ – mid-15^th^ centuries), where some of currently known caves were used for economic and religious purposes. After the siege and capture of Dori in 1475 by Ottoman troops, the Turkish fortress of Mangup-Kale was built on the plateau and existed there until 1774. In our opinion, under the influence of long-term human activity on Baba-Dagh Plateau, special conditions were created for the colonisation of caves by troglophilous species, which could have been unintentionally introduced to Crimea by active trade between the medieval Principality of Theodoro and/or the Turkish fortress of Mangup-Kale and medieval states of the west Caucasus and the Ottoman Empire (Herzen and Makhneva-Chernets 2006), the native range of *A.borutzkyi* lays (Nadolny and Kovblyuk 2007). Therefore, in Crimea *A.borutzkyi* seems to be an accidentally introduced facultative synanthrope, locally established in suitable subterranean biotope as a subtroglophile.

###### Additional diagnostic details.

**Figure 15.**
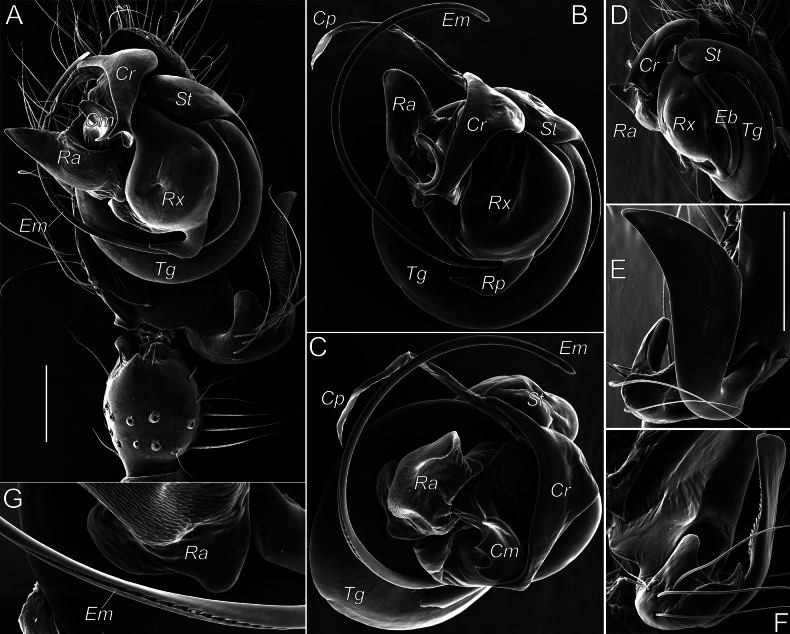
Male palp of *Aituariaborutzkyi* from Mangupskaya I Cave: **A** palp, ventral view; **B–D** bulbus, ventral, anterior, and retrolateral views **E, F** paracymbium, dorso-retrolateral and ventro-retrolateral views **G** fragment of embolus with a furrow, prolateral view. Abbreviations: *Cm* – median process of the conductor, *Cp* – prolateral process of the conductor, *Cr* – retrolateral process of the conductor, *Eb* – embolic base, *Em* – embolus, *Ra* – radical apophysis, *Rp* – radical process, *Rx* – radix, *St* – subtegulum, *Tg* – tegulum. Scale bars: 0.2 mm (**A, E**); not scaled (**B–D, F, G**).

**Figure 16.**
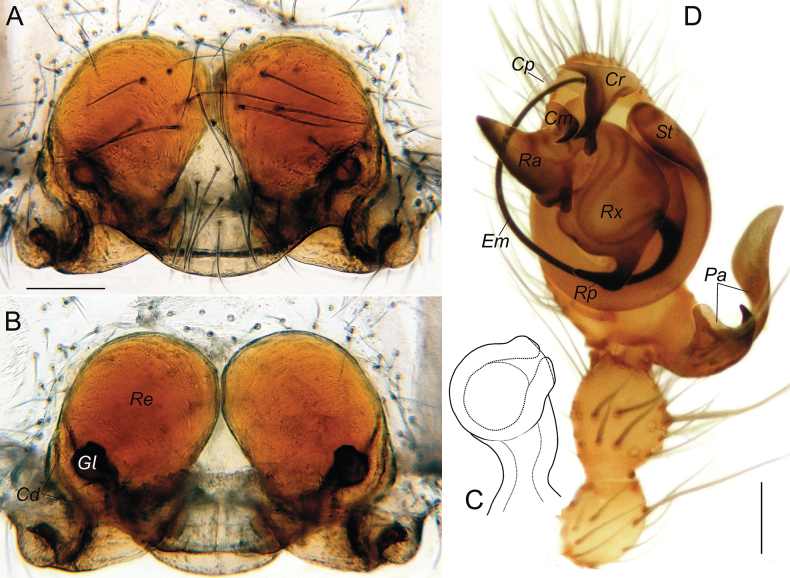
Female and male copulatory organs of *Aituariaborutzkyi* from Mangupskaya I Cave: **A, B** epigyne, ventral and dorsal views **C** club-like gland, dorsal view **D** male palp, ventral view. Abbreviations: *Cd* – copulatory duct, *Cm* – median process of the conductor, *Cp* – prolateral process of the conductor, *Cr* – retrolateral process of the conductor, *Em* – embolus, *Gl* – club-like gland, *Pa* – paracymbium, *Ra* – radical apophysis, *Re* – receptacle, *Rp* – radical process, *Rx* – radix, *St* – subtegulum. Scale bars: 0.2 mm (**A, B, D**); not scaled (**C**).

The complex structure of copulatory organs in *Aituaria* members has been discussed and illustrated (Marusik et al. 2017; Fomichev et al. 2022). Here we give SEM micrographs of the male palp of *A.borutzkyi* to show their details (Fig. 15A–G): the paracymbium bears three apophyses, of which the dorsal and distal apophyses are covered with triangular scales (Fig. 15A, E, F); the rounded anterior part of subtegulum is prominent in ventral view, the rest of it is hidden behind the tegulum (Fig. 15A); the tegulum is discoidal; the radix pear-shaped with a pointed posterior process; the triangular radical apophysis has a scaly surface (Fig. 15A, C, G); the conductor consists of three parts: median process with two pointed and one rounded outgrowths, the relatively massive retrolateral process that is bifurcated on its ventral side, and the long, narrow and transparent prolateral process (Fig. 15B, C); the connection between embolus and tegulum forms a sharp bend on the anterio-retrolateral side of the bulb (Fig. 15D); the embolus with a furrow almost along its entire length (Fig. 15C, G). For the female of *A.borutzkyi*: the epigyne has a rounded edge and is oval, with poorly sclerotised receptacles; the spherical, club-like gland is heavily sclerotised (Fig. 16A, B); the copulatory openings situate near the epigynal edge, entering the receptacles laterally (Fig. 16B).

A recent review (Fomichev et al. 2022) considered four species of *Aituaria*, of which only two have been found in Crimea, including its subterranean habitats: viz., *A.borutzkyi* and *A.pontica*. Males of these species are easily separable by the embolic shape: the narrow embolus in *A.borutzkyi* and wide in *A.pontica*; also, all other apophysis differ in their shapes (cf. Fomichev et al. 2022: figs 25, 27). The females differ in detailed structures of the epigyne (cf. Fig. 16A–C and Marusik et al. 2017: figs 18, 19): *A.pontica* has a more bended edge of the epigynal plate compared to that in *A.borutzkyi*; the stem and head in the club-shaped gland are of equal width in *A.pontica*, while the head is wider than the stem in *A.borutzkyi*.

##### ﻿ Aituaria pontica

Taxon classificationAnimaliaAraneaeNesticidae

(Spassky, 1932)

1A52DEA6-6BCB-5F88-8C7E-AEBD8F44FE56

Fig. 2A

Aituaria pontica (Spassky, 1932): Nadolny and Turbanov 2014: 569; Kovblyuk and Kastrygina 2015: 42; Turbanov et al. 2016b: 1284; Esyunin 2017: 243; Turbanov and Nadolny 2017: 114–115.

###### Material examined.

• 2 ♂♂, 1 ♀, 5 juv. (TNU), Crimea, nr Sevastopol, Khomutovaya Gorge, Maksimova Datsha, abandoned aqueduct carved into an unnamed cave-spring, 11.III.2014, I.S. Turbanov leg.

###### Distribution.

It is found in natural habitats in Krasnodar Territory, Russia. Also, reported from Ukraine and Russia (the Urals) as a synanthropic species (Nadolny and Turbanov 2014; Esyunin 2017).

###### Records from the Crimean caves.

**Figure 17.**
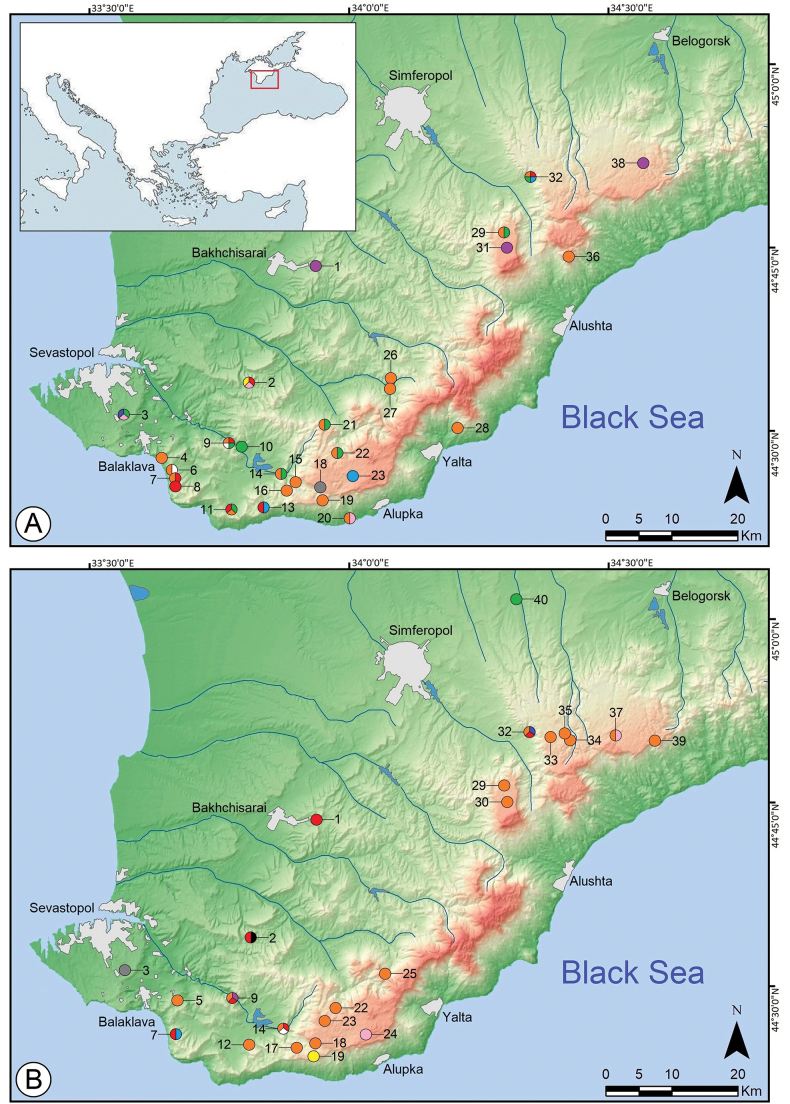
Distributions of cave-dwelling spiders in Crimea (including data from references and this work): **A***Tegenarialapicidinarum* (purple circle), *T.parietina* (blue circle), *T.taurica* (orange circle), *Amaurobiuserberi* (yellow circle), *Alopecosafarinosa* (grey circle), *Pholcusphalangioides* (pink circle), *Metabourneti* (red circle), *Metellinasegmentata* (pale blue circle), *M.merianae* (green circle), *Steatodatriangulosa* (white circle) **B***Bisetifergruzin* (blue circle), *B.tactus* sp. nov. (purple circle), *Caviphantesdobrogicus* (green circle), *Lepthyphantesleprosus* (red circle), *Megalepthyphantesnebulosus* (pale blue circle), *M.pseudocollinus* (white circle), *Palliduphanteskhobarum* (orange circle), ? *Tenuiphanteszimmermanni* (pink circle), *Troglohyphantesexspectatus* sp. nov. (yellow circle), *Aituariapontica* (grey circle), *A.borutzkyi* (black circle). The numbering of the caves is common for both maps: 1 – in unnamed cave near the city of Bakhchisarai; 2 – Mangupskaya I (= MK-1) Cave; 3 – abandoned aqueduct carved into an unnamed cave-spring in area of the Maksimova Datsha; 4 – Asketi I Cave; 5 – Kay-Kobasy Cave; 6 – Izumrudnaya Сave; 7 – Gekkonovaya Cave; 8 – Gnomov Cave; 9 – Tshernoretshenskaya Cave; 10 – Azis-Koba (= Kara-Koba) Cave; 11 – Mamut-Tshokrak Cave; 12 – Baidar-Tshokrak Cave; 13 – unnamed cave on northeastern slope of Mt. Kilse-Burun; 14 – Skelskaya Cave; 15 – Rodnikovskaya Cave; 16 – Koryta (= Kuznetsova) Cave; 17 – Zemlyanitshnaya Cave; 18 – Kristalnaya (= Maksimovitcha) Cave; 19 – Druzhba Cave; 20 – unnamed cave (= ?Malaya Cave) in Nizhnie Limeny (now Goluboi Zaliv); 21 – Daniltsha-Koba Cave; 22 – Ayu-Teshik Cave; 23 – Villyaburunskaya Cave; 24 – Troizkogo (= Kharkovskaya, ZUG) Сave; 25 – Avantyura Cave; 26 – Kaply-Kayanskaya (= Khaplu-Khoba) Cave; 27 – Ayu-Koba Cave; 28 – grotto in Massandra; 29 – Binbash-Koba Cave; 30 – Paskhalnaya Cave; 31 – Alushtinskaya Cave; 32 – Kizil-Koba (= Krasnaya) Cave; 33 – Sliyanie Cave; 34 – Vostotshny Potok Cave; 35 – Partizanskaya Cave; 36 – grotto on Mt. Yuznaya Demerdzhi; 37 – Tisovaya Cave; 38 – Karani-Koba Cave; 39 – Tuakskaya (= Ful-Koba) Cave; 40 – Tavrida Cave.

Map (Fig. 17B – grey circle). Abandoned aqueduct carved into an unnamed cave-spring of Maksimova Datsha nr Sevastopol (Nadolny and Turbanov 2014).

###### Ecology.

A troglophile and synanthropic species (Esyunin 2017). In Crimea, the species has been found only in an abandoned aqueduct in Sevastopol, which was made by enlarging a cave spring (Nadolny and Turbanov 2014). Maksimova Datsha was the site with intensive agricultural and other economic activities carried out in the second half of the 19^th^ and early 20^th^ centuries, where ornamental/cultivated plants were introduced mainly from the west Caucasus (Chikin 2005). In our opinion, this was a pathway for spreading alien species to Crimea, including *A.pontica*. Therefore, in Crimea this species is an accidentally introduced facultative synanthrope, locally established in suitable subterranean biotope as a subtroglophile.

### ﻿Family Pholcidae C.L. Koch, 1850

#### ﻿Genus *Pholcus* Walckenaer, 1805

##### ﻿ Pholcus phalangioides

Taxon classificationAnimaliaAraneaePholcidae

(Fuessling, 1775)

8E90EFAB-5518-5ECF-A763-4400847350DE

Pholcus phalangioides (Fuessling, 1775): Charitonov 1947a: 47; Charitonov 1947b: 1; Birstein 1963: 128; Tyshchenko 1971: 23; Kovblyuk 2004a: 238; Kovblyuk 2014: 44; Turbanov et al. 2016b: 1283.
Pholcus phalangoides [sic!] (Fuessling, 1775): Evtushenko 2004: 66, 68.
Pholcus phalangoidaes [sic!] (Fuessling, 1775): Zagorodniuk and Vargovitsh 2004: 207.

###### Material examined.

• 1 ♀ (TNU 10193/1), Crimea, nr Sevastopol, Khomutovaya Gorge, Maksimova Datsha, abandoned aqueduct carved into an unnamed cave-spring, 11.III.2014, I.S. Turbanov leg. • 1 ♂ (TNU 10190/3), same cave, 23.V.2015, A.A. Nadolny leg. • 1 ♂ (TNU), Crimea, Bakhchisarai Distr., nr Khodzha-Sala Vil., steep southern slope of Baba-Dagh Plateau (= Mangup-Kale Gorodishche), Mangupskaya I (= MK-1) Cave, 2.VI.2021, I.S. Turbanov leg.

###### Distribution.

Cosmopolite (Kovblyuk and Kastrygina 2015; Nentwig et al. 2024).

###### Records from the Crimean caves.

Map (Fig. 17A – pink circle). Small unnamed cave (= ?Malaya Cave) in Nizhnie Limeny (now Goluboi Zaliv, Yalta) and abandoned aqueduct of Maksimova Datsha nr Sevastopol; Mangupskaya I (= MK-1) Cave in Bakhchisarai Distr. (Charitonov 1947a; present data).

###### Ecology.

A troglophile and synanthropic species (Mammola et al. 2018; Nentwig et al. 2024). *Pholcusphalangioides* usually is found in anthropogenic biotopes and less frequently in caves (Huber 2011). In Crimea, this species is also mainly synanthropic (Kovblyuk et al. 2016), except for few findings in caves (Charitonov 1947a; present data), which are somehow associated with human economic activity. In particular, in the abandoned aqueduct in Sevastopol, which was made by enlarging a cave spring, and where intensive agricultural and other economic activities were carried out in the second half of the 19^th^ and early 20^th^ centuries (Chikin 2005). Small unnamed cave in Nizhnie Limeny (Charitonov 1947a) is another site from where *P.phalangioides* has been recorded. That site is situated on Koshka Mt. containing the ruins of a medieval Genoese fortification (shelter) from the 13–15^th^ centuries, Limena-Kale (Myts 1991). Therefore, in Crimea, this species can be classified as a facultative synanthrope, established as a subtroglophile in suitable subterranean biotopes.

### ﻿Family Tetragnathidae Menge, 1866

#### ﻿Genus *Meta* C.L. Koch, 1836

##### Meta bourneti

Taxon classificationAnimaliaAraneaeTetragnathidae

Simon, 1922

B19DBE21-57A6-53C2-8DA2-88AFE5752E46

Fig. 2E–G

Meta bourneti Simon, 1922: Spassky 1936: 535; Charitonov 1936: 201; Charitonov 1939: 197; Charitonov 1947a: 44–45; Charitonov 1947b: 1; Birstein 1963: 128; Tyshchenko 1971: 190; Mikhailov 1997: 108; Amelichev et al. 2004: 136; Evtushenko 2004: 66, 68; Zagorodniuk and Vargovitsh 2004: 207; Kovblyuk 2004a: 244; Mikhailov 2013: 101; Kovblyuk 2014: 44–45, fig. 23; Kovblyuk and Kastrygina 2015: 56; Turbanov et al. 2016b: 1283; Prokopov and Turbanov 2017: 101; Turbanov et al. 2019a: 218; Samokhin et al. 2019: 247.
Meta sp.: Turbanov et al. 2019b: 41.

###### Material examined.

• 1 ♀ (TNU 10265), Crimea, Bakhchisarai Distr., nr Khodzha-Sala Vil., steep southern slope of Baba-Dagh Plateau (= Mangup-Kale Gorodishche), Mangupskaya I (= MK-1) Cave, 6–8.V.2017, O.L. Makarova, K.V. Makarov leg. • 2 ♂♂ (TNU 10191/2) • 2 ♀♀ (IT), same cave, 2.X.2020, I.S. Turbanov, A.A. Turbanova leg. • 1 ♂, 1 ♀, 4 juv. (IT), same cave, 2.VI.2021, I.S. Turbanov leg. • 1 ♂ (TNU 10179), Crimea, Sevastopol, nr Balaklava, Aya Cape Mt. Range, Mt. Kala-Fatlar, Gnomov Cave, 9.X.2016, A.A. Nadolny leg. • 1 ♂ 1 ♀ (IT), Crimea, Sevastopol, nr Balaklava, Aya Cape Mt. Range, Kala-Fatlar Mt., Gekkonovaya Cave, 28.XII.2012, I.S. Turbanov leg. • 1 ♀ (TNU 10229) • 3 juv. (IT), Crimea, nr Sevastopol, Tshernaya River canyon, Tshernoretshenskaya Cave, 5.V.2017, I.S. Turbanov leg. • 1 ♂ (TNU 10231/1), same cave, 3.III.2018, A.A. Nadolny leg. • 1 ♀ (TNU 10287/3) • 2 ♀♀ 3 juv. (IT), same cave, 3.VI.2021, I.S. Turbanov, A.A. Nadolny leg. • 1 ♀ (TNU 10191/2), Crimea, nr Sevastopol, northeastern slope of Baidarskаya Yaila, Baidarskaya Valley, nr Kizilovoye Vil., Mamut-Tshokrak Cave, 1–2.VI.2013, I.S. Turbanov leg. • 1 ♂ (TNU 10228), same cave, 25.VI.2017, I.S. Turbanov leg. • 2 ♀♀ (TNU 10181/1), Crimea, nr Sevastopol, western part of Ai-Petri Yaila, northeastern slope of Kilse-Burun Mt., unnamed cave, 14.IV.2014; I.S. Turbanov leg • 1 ♂, 1 ♀ (IT), Crimea, Simferopol Distr., nr Perevalnoye Vil., western slope of Dolgorukovskaya Yaila, Kizil-Koba (= Krasnaya) Cave, 18.XII.2019, I.S. Turbanov leg.

###### Distribution.

West Palearctic nemoral-subtropical: from Portugal to Georgia and from Britain to North Africa (Kovblyuk and Kastrygina 2015; Nentwig et al. 2024).

###### Records from the Crimean caves.

Map (Fig. 17A – red circle). Caves in the south-western and central parts of the Crimean Mountains: Mangupskaya I, Gnomov, Gekkonovaya, Tshernoretshenskaya, Mamut-Tshokrak, unnamed cave on the northeastern slope of Kilse-Burun Mt. and Kizil-Koba (Spassky 1936; Charitonov 1947a; Evtushenko 2004; Turbanov et al. 2019a, b; present data).

###### Ecology.

A troglophile (Mammola et al. 2018). In Crimea, *M.bourneti* has been recorded only from caves, and hence is classified as a eutroglophile. There are data on the life cycle of *M.bourneti*, according to which the first instars of its postembryonic development may occur outside of caves (Mammola and Isaia 2014). However, we have repeatedly recorded juvenile specimens of different instars in Mangupskaya I and Tshernoretshenskaya caves, including those found at 50–100 meters from the entrances, and this suggests that in Crimea the life cycle of *M.bourneti* is completely restricted to caves.

###### Remarks.

The reference of *Meta* sp. for the caves of Cape Aya (Turbanov et al. 2019b) refers to the material from Gnomov and Gekkonovaya caves used in present data.

#### ﻿Genus *Metellina* Chamberlin & Ivie, 1941

##### ﻿ Metellina merianae

Taxon classificationAnimaliaAraneaeTetragnathidae

(Scopoli, 1763)

65C01FCF-0992-5018-B3E0-1E69734C697E

Fig. 2C, D

Meta merianae (Scopoli, 1763): Spassky 1927: 4; Charitonov 1932: 123; Charitonov 1939: 197; Charitonov 1947a: 45–46; Charitonov 1947b: 1; Birstein 1963: 128; Tyshchenko 1971: 23.
Metellina merianae (Scopoli, 1763): Amelichev et al. 2004: 136; Evtushenko 2004: 66, 68; Kovblyuk 2004a: 245; Zagorodniuk and Vargovitsh 2004: 207; Turbanov et al. 2016b: 1283; Prokopov and Turbanov 2017: 101; Samokhin et al. 2019: 247; Turbanov et al. 2019a: 218.

###### Material examined.

• 5 ♀♀ (TNU 10193/2), Crimea, nr Sevastopol, Khomutovaya Gorge, Maksimova Datsha, abandoned aqueduct carved into an unnamed cave-spring, 11.III.2014, I.S. Turbanov leg. • 1 ♀ (TNU 10190/2), same cave, 23.V.2015, A.A. Nadolny leg. • 1♀ (TNU 10287/4), Crimea, nr Sevastopol, canyon of the Tshernaya River, Tshernoretshenskaya Cave, 3.VI.2021, I.S. Turbanov, A.A. Nadolny leg. • 1 ♀ (TNU 10184), Crimea, nr Sevastopol, northeastern slope of Baidarskаya Yaila, Baidarskaya Valley, nr Kizilovoye Vil., Mamut-Tshokrak Cave, 10.VIII.2010, I.S. Turbanov leg. • 1 ♂ 1 ♀ (TNU 10191/1), same cave; 1–2.VI.2013, I.S. Turbanov leg. • 1 ♀ (TNU 10238/2), Crimea, nr Sevastopol, northwestern slope of Ai-Petri Yaila, Baidarskaya Valley, nr Rodnikovskoye Vil., entrance to Skelskaya Cave, 25.IX.2018, I.S. Turbanov, A.A. Turbanova leg. • 1 ♀ (IT), same cave, 27.XII.2019, I.S. Turbanov leg. • 2 ♂♂ 6 ♀♀ (IT), same cave, 1.VI.2021, I.S. Turbanov, A.A. Turbanova leg. • 1 ♀ (TNU 10261/2), Crimea, Bakhchisarai Distr., northern part of Ai-Petri Yaila, Ayu-Teshik Mt., Ayu-Teshik Cave, 8.V.2015, I.S. Turbanov leg. • 1 ♂ 1 ♀ (TNU 10258), same cave, 16.VII.2017, O.V. Kukushkin leg • 4 ♂♂ (TNU 10195/2), Crimea, Simferopol Distr., northern part of Tshatyr-Dagh Yaila, Binbash-Koba Cave, 12.II.2015, I.S. Turbanov leg.

###### Distribution.

West and Central Palaearctic polyzonal: from Portugal to the Altai Mts and from Scandinavia to Iran (Kovblyuk and Kastrygina 2015; Nentwig et al. 2024).

###### Records from the Crimean caves.

Map (Fig. 17A – green circle). Caves of the southwestern and central parts of the Crimean Mountains: Tshernoretshenskaya, Azis-Koba (= Kara-Koba), Mamut-Tshokrak, Skelskaya, Ayu-Teshik, Daniltsha-Koba, Binbash-Koba, and Kizil-Koba, as well as abandoned aqueduct of Maksimova Datsha nr Sevastopol (Spassky 1927; Charitonov 1947a; Samokhin et al. 2019; Turbanov et al. 2019a; present data).

###### Ecology.

A troglophile (Mammola et al. 2018). In Crimea, the species inhabits broad-leaved forests of northern macro-slopes of the mountains (Kovblyuk and Kastrygina 2015). In the Crimean caves, *M.merianae* can be classified as a subtroglophile.

##### ﻿ Metellina segmentata

Taxon classificationAnimaliaAraneaeTetragnathidae

(Clerck, 1757)

CEEF2C79-1E1C-50B0-BC5E-0E1A35B6B64E

###### Material examined.

• 1 ♀ (TNU 10181/2), Crimea, nr Sevastopol, the western part of Ai-Petri Yaila, the northeastern slope of Kilse-Burun Mt., unnamed cave, 14.IV.2014; I.S. Turbanov leg. • 1 ♀ (TNU 10194), Crimea, Bakhchisarai Distr., the central part of Ai-Petri Yaila, Vorontsovsky Forest, Rutsheinaya Cave, 8–9.II.2014, I.S. Turbanov leg. • 1 ♂ (TNU 10178), Crimea, Simferopol Distr., nr Perevalnoye Vil., the western slope of Dolgorukovskaya Yaila, Kizil-Koba (= Krasnaya) Cave, 8–9.XI.2014, A.A. Nadolny leg.

###### Distribution.

Transpalaearctic polyzonal (Kovblyuk and Kastrygina 2015; Nentwig et al. 2024).

###### Records from the Crimean caves.

Map (Fig. 17A – pale blue circle). Unnamed cave on the northeastern slope of Kilse-Burun Mt., Rutsheinaya Cave on Ai-Petri Yaila and Kizil-Koba Cave on the western slope of Dolgorukovskaya Yaila (present data).

###### Ecology.

In Crimea, *M.segmentata* is common in the mountainous forest part of the Peninsula (Kovblyuk and Kastrygina 2015). There is an indication of this species as a trogloxene in Tshudesnitsa Cave in Perm Oblast of Russia (Pankov et al. 2009). In the Crimean caves, *M.segmentata* can be classified as a trogloxene.

### ﻿Family Theridiidae Sundevall, 1833

#### ﻿Genus *Steatoda* Sundevall, 1833

##### ﻿ Steatoda triangulosa

Taxon classificationAnimaliaAraneaeTheridiidae

(Walckenaer, 1802)

1959401C-4547-5FAB-B670-48AADF843DDB

###### Material examined.

• 1 ♀ (TNU 10259/2), Crimea, Sevastopol, nr Balaklava, Aya Cape Mt. Range, Kala-Fatlar Mt., Izumrudnaya Сave, 20.III.2016, O.V. Kukushkin leg. • 1 ♀ (TNU 10287/5), Crimea, nr Sevastopol, Tshernaya River canyon, entrance to Tshernoretshenskaya Cave, 3.VI.2021, I.S. Turbanov, A.A. Nadolny leg.

###### Distribution.

Cosmopolite (Kovblyuk and Kastrygina 2015; Nentwig et al. 2024).

###### Records from the Crimean caves.

Map (Fig. 17A – white circle). Izumrudnaya Cave on Kala-Fatlar Mt. of the Aya Cape Mt. Range and Tshernoretshenskaya Cave nr Sevastopol (present data).

###### Ecology.

A troglophile and synanthropic species (Nentwig et al. 2024). In Crimea, it has been recorded everywhere as a synanthropic, except for the southern coast where it occurs in natural habitats (Kovblyuk and Kastrygina 2015). In the Crimean caves, the ecological association of *S.triangulosa* remains unclear. Since we have collected only two specimens from the entrances of Tshernoretshenskaya and Izumrudnaya caves, it is likely to be a subtroglophile.

## ﻿Discussion

A total of 20 spider species in eight families have been discovered in the Crimean caves. Of these species, four have Crimean caves as their type locality: viz., *Tegenariataurica* and *Palliduphanteskhobarum* (see Charitonov 1947a), *Bisetifertactus* sp. nov., and *Troglohyphantesexspectatus* sp. nov. The dubious record of ?*Tenuiphanteszimmermani* (Evtushenko 2004; Zagorodniuk and Vargovitsh 2004) is not taken into account, as it requires confirmation.

According to the literature-derived and present data, spiders have been recorded in 40 caves of the Crimean Mountains (see Fig. 17), accounting for just 2.5% of all the known karst cavities (Amelichev et al. 2014). The highest spider diversity was recorded in Tshernoretshenskaya and Kizil-Koba Caves, each with seven species (Fig. 17).

Based on the study of local populations, cave spiders in Crimea are classified into four ecological groups:

1. Troglobionts – a single species, *Bisetifertactus* sp. nov. It has clear troglomorphic features, such as the almost completely reduced eyes. This is only the second true troglobiont spider species with reduced eyes from the caves of the former USSR; the first was *Iberina* (?) *ljovuschkini* Pichka, 1965 found in Shakalya Cave (the West Caucasus, Russia) (Pichka 1965), currently considered a nomen dubium (Růžička 2022);
2. Eutroglophiles – six species, *Tegenariataurica*, *Bisetifergruzin*, *Caviphantesdobrogicus*, *Palliduphanteskhobarum*, *Troglohyphantesexspectatus* sp. nov., and *Metabourneti*. They do not possess noticeable troglomorphic features, but in Crimea they are confined to caves only and/or are capable of maintaining stable subterranean populations;
3. Subtroglophiles – nine species. They can be subdivided into three groups: (i) native subtroglophilous species: *Tegenarialapicidinarum*, *Lepthyphantesleprosus*, *Megalepthyphantesnebulosus*, *Metellinamerianae*, and *Steatodatriangulosa*, which are known from both caves and epigeic habitats, repeatedly reported by other researchers as troglophiles; (ii) cosmopolitan subtroglophilous species: *Tegenariaparietina* and *Pholcusphalangioides*, which in Crimea are not a native but rather facultative synanthropic species, because they occur in the caves that have been used for economic human activities; (iii) subtroglophiles unintentionally introduced: *Aituariaborutzkyi* and *A.pontica*, locally established as facultative synanthropes in caves that were used for economic human activities;
4. Trogloxenes – four species, *Amaurobiuserberi*, *Megalepthyphantespseudocollinus*, *Alopecosafarinosa*, and *Metellinasegmentata*. They accidentally appear in caves, since their life cycles are not associated with subterranean biotopes; they have not been reported for subterranean biotopes previously, or indicated by other researchers as trogloxenes in other parts of their ranges.

In his review, Charitonov (1947a) characterised the araneofauna of the Crimean caves as “*Tegenaria*–*Lephthyphantes*” (under the name *Lephthyphantes* was meant *Palliduphanteskhobarum*) and contrasted it with the araneofauna of Caucasus caves, which he termed “*Nesticus*–*Troglohyphantes*” (under the name *Nesticus* were meant various members of the family Nesticidae). As another important feature, he considered the Crimean cave araneofauna conflicted with the data on other arthropod groups (because no troglobiont spiders were found), and only further study of the Crimean caves would be able to change this situation or give an opportunity to explain it correctly. Thus, based on the review of the cave biota of the former USSR (Turbanov 2016a, b, cc) and taking into account more recent taxonomic works (Golovatch et al. 2017; Vinarski and Palatov 2019; Sendra et al. 2020; Turbanov and Kolesnikov 2020, 2021; Marin et al. 2022), the Crimean caves have most likely acted as glacial refugia for many of troglo- and stygomorphic invertebrates, as suggested by more than 50 reported species, including mainly crustaceans, pseudoscorpions, millipedes, diplurans, springtails, and beetles. Yet, Kovblyuk (2014) pointed out the low species diversity and lack of endemics of the araneofauna of the Crimean caves. As an explanation, he suggested that the ancient cave fauna became extinct during marine transgressions and karst flooding, and that the modern cave fauna consists of species that have colonised the peninsula relatively recently. Based on the new data, it seems possible to partially answer the questions raised by Charitonov (1947a) and Kovblyuk (2014) and thereby in general provide a possible reconstruction of the genesis of the araneofauna of the Crimean caves.

The bulk, 80%, of the spider species considered are widespread, with cosmopolitan, Holarctic, trans-Palaearctic, West and Central Palaearctic, East European or East Mediterranean ranges: *Aituariaborutzkyi*, *A.pontica*, *Alopecosafarinosa*, *Caviphantesdobrogicus*, *Tegenarialapicidinarum*, *T.parietina*, *Amaurobiuserberi*, *Lepthyphantesleprosus*, *Metabourneti*, *Megalepthyphantesnebulosus*, *M.pseudocollinus*, *Metellinamerianae*, *M.segmentata*, *Palliduphanteskhobarum*, *Pholcusphalangioides*, *Steatodatriangulosa*. At present, we have no data for these species to establish the chronology of their colonisation across Crimea. It is possible that the scenario indeed conforms to Kovblyuk’s (2014) speculation that most species colonised Crimea during the multiple Pleistocene-Holocene regressions of the Black Sea basin, when shelf zoogeographic corridors between Crimea, the Caucasus and the Balkans opened up. However, we believe that four synanthropic species – *Aituariaborutzkyi*, *A.pontica*, *Tegenariaparietina*, and *Pholcusphalangioides* – entered the Crimean caves during historical times Further considerations on the history of the cave araneofauna will be based on analysing the distribution of species with restricted ranges: viz., the three Crimean endemics, *Tegenariataurica*, *Bisetifertactus* sp. nov., and *Troglohyphantesexspectatus* sp. nov., and the Crimean-Caucasian subendemic *Bisetifergruzin*.

The genesis of cave habitats is known to occur simultaneously with the formation of the caves themselves (Prokopov and Turbanov 2017). The recent relief of the Crimean Mountains was formed in the late Pliocene and Pleistocene periods (Muratov and Nikolaev 1940; Muratov 1954; Vakhrushev 2010), which determined the predominantly Pleistocene age of the Crimean karst (Dublyansky 1966, 1977; Vakhrushev 2010; Klimchouk et al. 2009, 2012; Amelichev et al. 2016). It is assumed that global climate changes associated with glacial periods and interglacials initiated colonisation of karst cavities (Jeannel 1959; Vandel 1964). Most likely, the terrestrial troglobiont fauna could have originated from forest litter dwellers. Cold periods, the development of ice shield, even locally, could have led a number of species to shelter in karst cavities, where the temperature regime was more stable (Jeannel 1965). Presumably the ancestral forms of the eutroglophilic spiders *Tegenariataurica* and *Troglohyphantesexspectatus* sp. nov. and the troglobiont spider *Bisetifertactus* sp. nov. were more widely distributed.

The species that are most morphologically related to *Tegenariataurica* have relatively large ranges in the Western Palaearctic: *T.ferruginea* (Panzer, 1804), *T.lapicidinarum*, and *T.parietina* (Nentwig et al. 2024). The current distribution of *T.taurica* in the Crimean Mountains is limited to the caves of the western and central karst massifs (see Fig. 17A – orange circle), which finally lost their geological and hydrological connections with each other in the late Pleistocene (Klimchouk 2009). This seems to indicate that, under the influence of global climate change, there has been a relatively recent (the late Pleistocene – early Holocene) simultaneous penetration of ancestral epigeic species in these isolated karst massifs. Furthermore, the caves of Montenegro on the Balkan Peninsula harbour a local endemic eutroglophile *T.gordani* Komnenov, 2020, which is morphologically very close to *T.taurica* (see Komnenov 2020). Their close relationships and restricted distribution (both are local endemics) are likely to reflect similar scenarios of their origin from a common widespread ancestor at the same time (late Pleistocene).

The genus *Bisetifer* has the Crimean-Caucasian range (Tanasevitch et al. 2015). In the Caucasus, both *Bisetifer* species inhabit humid epigeic microbiotopes (Tanasevitch 1987; Tanasevitch et al. 2015). The common to Crimea and the Caucasus, *B.gruzin* occurs only in the Crimean caves, which, in our opinion, could indicate its recent (probably the early Holocene) zoogeographic connections. Yet, due to the drier climate of Crimea in the late Holocene, this species appears to have used caves as more mesophilic habitats, while its epigeic populations may have become extinct.

According to Isaia et al. (2017), the large genus *Troglohyphantes* has an ancient Mediterranean origin and some of its species occur in epigeic habitats. Thus, *T.exspectatus* sp. nov. is morphologically closest to the Balkan local endemic troglobiont *T.drenskii*, as well as to the Caucasian troglophile *T.deelemanae* and the epigeic *T.adjaricus*, which may indicate the ancient (Pleistocene) Eastern Mediterranean biogeographical connections in this genus.

Both newly described species, *B.tactus* sp. nov. and *T.exspectatus* sp. nov., have narrow distribution and are known only from their type localities. The microclimatic conditions of the caves where the new species were discovered seems to be unique for surviving some species, making these caves a kind of Pleistocene refugia.

**Figure 18.**
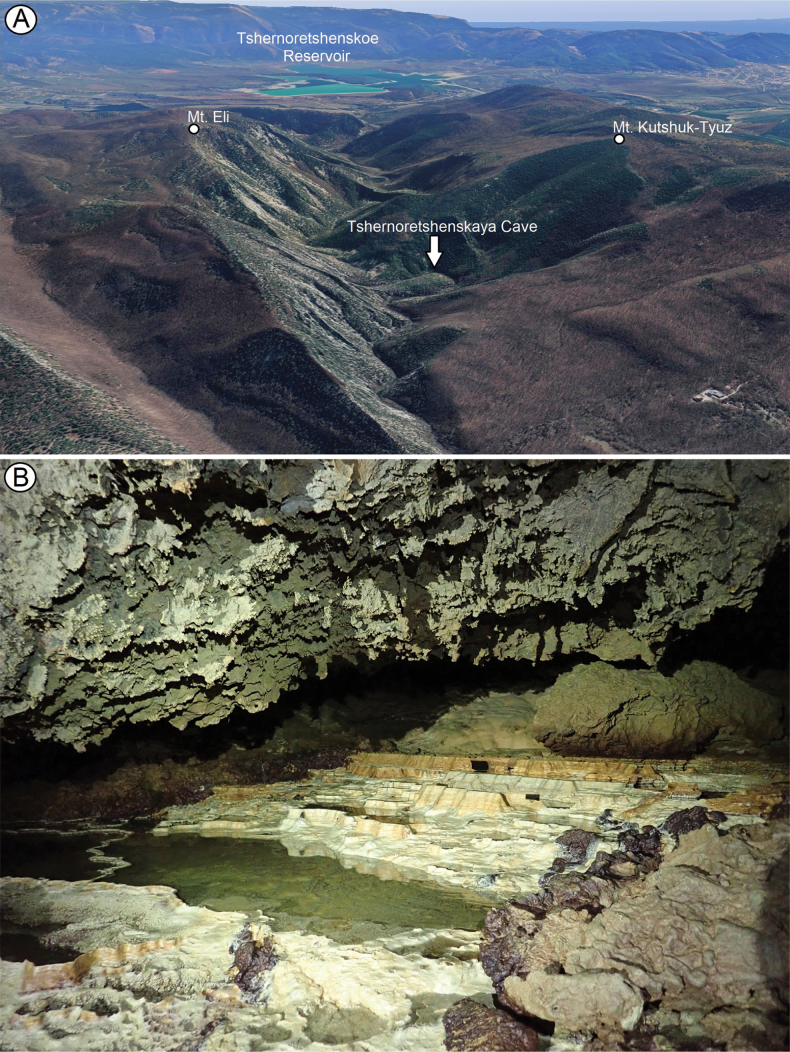
Location and biotope of the type locality of *Bisetifertactus* sp. nov.: **A** map with type locality showing the entrance of Tshernoretshenskaya Cave (from Google Earth Pro) **B** biotope inhabited by the new species in Tshernoretshenskaya Cave (photographs by IST).

*Bisetifertactus* sp. nov. is described from the small horizontal Tshernoretshenskaya Cave (length 87 m) situated in the central part of the Tshernaya River canyon, the south-western Crimea (see Fig. 18). This is the only known cave in the canyon with stable microclimatic conditions – high relative humidity and a very static annual temperature in the range from 10.5 °С to 12.4 °С (present data). Such uniquely stable conditions are possible due to a small entrance and the presence of a permanent small watercourse (see Fig. 18B). All other caves of the Tshernaya River Canyon differ from Tshernoretshenskaya Cave in having large entrances, shorter lengths, lacking watercourses, and higher seasonal fluctuations in relative air humidity and temperatures: e.g., in Tomenko Cave from 2 °С to 21 °С, Azis-Koba (= Kara-Koba) Cave from 4 °С to 17 °С (present data).

**Figure 19.**
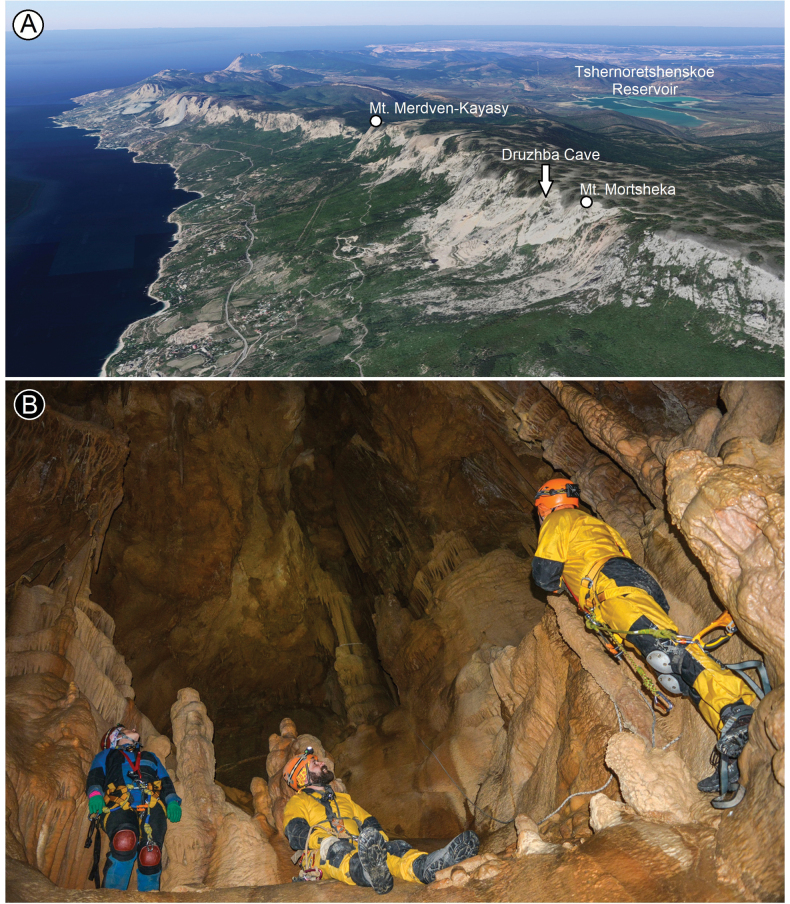
Location and biotope of the type locality of *Troglohyphantesexspectatus* sp. nov. **A** map with type locality showing the entrance of Druzhba Cave (from Google Earth Pro) **B** biotope inhabited by the new species in Druzhba Cave (photographs by Yu.S. Balakhtinova).

*Troglohyphantesexspectatus* sp. nov. is described from the small vertical Druzhba Cave (depth 45 m), which is located in the southern cliffs of Mortsheka Mt., the west part of Ai-Petri Yaila, the south-western Crimea (see Fig. 19). This is the only cave in the area at hand with stable microclimatic conditions: a high relative humidity and very static annual temperature ranging from 9.7 °С to 12.5 °С (present data). These unique conditions are possible due to cave structure as well: the small entrance and the cave’s position on cliffs of the southern macroslope, which contributes to the warm climatic conditions of the site. In comparison, the horizontal Honey Cave is nearby on the same slope but due to its huge entrance, it experiences seasonal fluctuations in relative humidity and temperature from 2.3 °С to 12.2 °С (present data). There are several vertical caves on the plateau of Mortsheka Mt., near Druzhba Cave. They also have relatively stable humidity, but because of the remoteness from the well-warmed edge of the southern cliff, the temperature inside them is lower: e.g., Akvalangistitsheskaya Cave, depth 80 m, temperatures range from 5.9 °С to 7.0 °С (present data). Thus, it seems possible to characterise *B.tactus* sp. nov. and *T.exspectatus* sp. nov. as stenobiont species inhabiting at relatively high humidity and rather static and comparatively high temperatures. The caves in which they occur are probably a refuge for Pleistocene thermophilic fauna.

## Supplementary Material

XML Treatment for Tegenaria lapicidinarum

XML Treatment for Tegenaria parietina

XML Treatment for Tegenaria taurica

XML Treatment for Amaurobius erberi

XML Treatment for Bisetifer gruzin

XML Treatment for Bisetifer tactus

XML Treatment for Caviphantes dobrogicus

XML Treatment for Lepthyphantes leprosus

XML Treatment for Megalepthyphantes nebulosus

XML Treatment for Megalepthyphantes pseudocollinus

XML Treatment for Palliduphantes khobarum

XML Treatment for Tenuiphantes zimmermanni

XML Treatment for Troglohyphantes exspectatus

XML Treatment for Alopecosa farinosa

XML Treatment for Aituaria borutzkyi

XML Treatment for Aituaria pontica

XML Treatment for Pholcus phalangioides

XML Treatment for Meta bourneti

XML Treatment for Metellina merianae

XML Treatment for Metellina segmentata

XML Treatment for Steatoda triangulosa

## ﻿Additional information

### Author ORCIDs

Anton A. Nadolny https://orcid.org/0000-0001-5555-1721

Ilya S. Turbanov https://orcid.org/0000-0001-9441-2791

### Data availability

All of the data that support the findings of this study are available in the main text.
